# A therapeutic cancer vaccine delivers antigens and adjuvants to lymphoid tissues using genetically modified T cells

**DOI:** 10.1172/JCI144195

**Published:** 2021-08-16

**Authors:** Joshua R. Veatch, Naina Singhi, Shivani Srivastava, Julia L. Szeto, Brenda Jesernig, Sylvia M. Stull, Matthew Fitzgibbon, Megha Sarvothama, Sushma Yechan-Gunja, Scott E. James, Stanley R. Riddell

**Affiliations:** 1 Clinical Research Division and Immunotherapy Integrated Research Center, Fred Hutchinson Cancer Research Center (FHCRC), Seattle, Washington, USA.; 2 Department of Medicine, University of Washington, Seattle, Washington, USA.; 3 Public Health Sciences Division, FHCRC, Seattle, Washington, USA.; 4 Memorial Sloan Kettering Cancer Center, New York, New York, USA.

**Keywords:** Immunology, Vaccines, Cancer immunotherapy

## Abstract

Therapeutic vaccines that augment T cell responses to tumor antigens have been limited by poor potency in clinical trials. In contrast, the transfer of T cells modified with foreign transgenes frequently induces potent endogenous T cell responses to epitopes in the transgene product, and these responses are undesirable, because they lead to rejection of the transferred T cells. We sought to harness gene-modified T cells as a vaccine platform and developed cancer vaccines composed of autologous T cells modified with tumor antigens and additional adjuvant signals (Tvax). T cells expressing model antigens and a broad range of tumor neoantigens induced robust and durable T cell responses through cross-presentation of antigens by host DCs. Providing Tvax with signals such as CD80, CD137L, IFN-β, IL-12, GM-CSF, and FLT3L enhanced T cell priming. Coexpression of IL-12 and GM-CSF induced the strongest CD4^+^ and CD8^+^ T cell responses through complimentary effects on the recruitment and activation of DCs, mediated by autocrine IL-12 receptor signaling in the Tvax. Therapeutic vaccination with Tvax and adjuvants showed antitumor activity in subcutaneous and metastatic preclinical mouse models. Human T cells modified with neoantigens readily activated specific T cells derived from patients, providing a path for clinical translation of this therapeutic platform in cancer.

## Introduction

Immune checkpoint inhibitors (ICIs) that target negative regulatory molecules on T cells have shown clinical efficacy in a subset of patients with multiple types of cancer (1, 2), highlighting the ability of endogenous T cells to eliminate tumors. Unfortunately, even in the tumor types most responsive to ICIs, a majority of the patients do not achieve durable tumor regression after such therapies. This may be due to a deficiency in the breadth and number of tumor-reactive T cells (3) or irreversible dysfunction of these responses (4–6), raising the possibility that vaccination to augment preexisting tumor-reactive T cells or elicit new responses might improve outcomes when combined with ICIs. It is acknowledged that cancer vaccines designed to augment T cell responses to cancer-specific self-antigens have had limited success (7). However, it is now feasible to vaccinate patients to elicit responses to multiple neoantigens, which are created by cancer-specific mutations and not subject to central tolerance mechanisms that can limit T cell avidity (8–10). Neoantigen-specific T cells have been shown to contribute to clinical responses after ICI therapy (11) and adoptive T cell transfer (12), therefore, augmenting these responses by vaccination may be beneficial.

Vaccination of patients with cancer is thought to be an important component of cancer immunotherapy. However, local administration of peptides, DNA, or recombinant viral vectors encoding tumor-associated antigens (TAAs) with adjuvants has mostly produced low-frequency T cell responses to these TAAs (7, 13), unlike effective antiviral immunity and adoptive T cell therapy for cancer, which achieve high frequencies of antigen-specific T cells in vivo (14–16). In murine models, recent vaccination strategies that deliver antigens systemically to lymphoid tissue and simultaneously activate innate immunity have induced stronger T cell responses, suggesting that systemic delivery might be more effective in patients (17, 18). This has been attempted using professional antigen-presenting cells (APCs) such as DCs to deliver tumor-associated neoantigens (19), but the difficulty of obtaining and manipulating large numbers of DCs and the variable biodistribution and survival of these cells after administration are significant limitations (20, 21).

Serendipitously, we observed that the adoptive transfer of gene-modified autologous human T cells that expressed a foreign protein induced robust and diverse endogenous CD8^+^ and CD4^+^ T cell responses specific for epitopes in the foreign protein, even in immunocompromised patients (22–24). These transgene product–specific T cell responses result in immune rejection of the transferred T cells and are prevented or delayed by the use of intensive lymphodepletion prior to adoptive transfer (22–24). Experiments in nonhuman primates in which autologous T cells were genetically modified and adoptively transferred also showed that immune responses were readily induced, even responses to a highly homologous protein that differed from the primate germline sequence by only a few amino acids (25), thus mimicking a neoantigen. T cells bearing highly immunogenic antigens were subsequently shown to elicit T cell responses in murine models, by a mechanism that involved cross-presentation of the antigen by endogenous DCs (26).

Adjuvants are used routinely to augment vaccination by providing inflammatory signals that activate DCs to induce costimulatory molecules and cytokines during priming of naive T cells (14). T cells are easily procured and can be readily expanded and genetically manipulated ex vivo, making it feasible to encode adjuvant signals in addition to tumor antigens in an autologous cell–based vaccine. Here, we genetically engineered T cells to express transgenes that encoded model antigens and immunostimulatory adjuvant signals and investigated their immunogenicity in preclinical models. We show that T cell–based vaccines can be designed to provide specific adjuvant signals and safely administered systemically to deliver antigens to DCs in lymphoid tissue. These vaccines induced potent antigen-specific CD8^+^ and CD4^+^ T cell responses that had antitumor activity in mice that was enhanced by combining autologous T cells modified with tumor antigens and additional adjuvant signals (Tvax) with immune checkpoint blockade (ICB) and other immunomodulators. Human T cells expressing neoantigen minigenes activated patient-derived neoantigen-specific T cells in vitro, indicating the potential for applying this approach in patients.

## Results

### Gene-modified T cells expressing model antigens (Tvax) prime CD8^+^ T cell responses.

**Figure 1:**
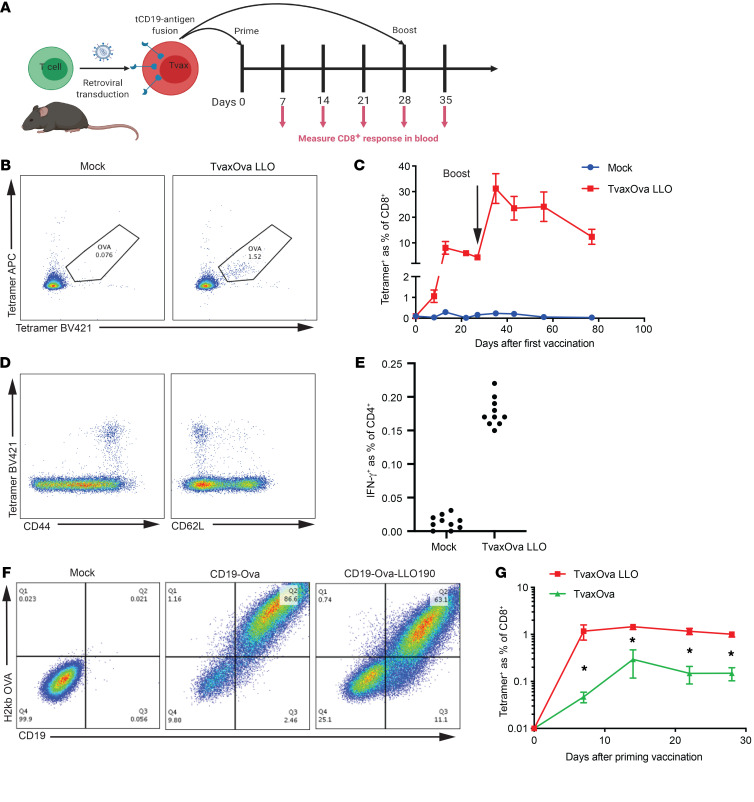
Tvax primes and boosts CD8^+^ T cell responses. (**A**) Schematic of syngeneic Tvax preparation and administration. (**B** and **C**) Frequency of OVA-specific CD8^+^ T cells in mice that received syngeneic Tvax. Cells were gated on CD8^+^ lymphocytes and stained with H-2Kb-SIINFEKL tetramers using 2 different fluorophores. Mice were injected on day 0 with Tvax cells transduced with retroviral constructs encoding tCD19 fused to an OVA CD8 epitope and an LLO190 CD4 epitope (Tvax_OVA-LLO190_). Control mice received mock-transduced T cells. OVA-specific T cells were detected after vaccination by staining with a tetramer and are expressed as a percentage of total CD8^+^ lymphocytes in the blood (*n =* 10 mice/group). (**D**) Staining for CD44 and CD62L and the OVA tetramer on CD8^+^ lymphocytes in peripheral blood 7 days after vaccination. (**E**) Frequency of CD4^+^IFN-γ^+^ T cells in spleens of vaccinated mice and control mice following restimulation with the LLO190 peptide 13 days after vaccination. (**F**) Expression of the H-2Kb-SIINFEKL epitope on Tvax cells expressing either the OVA CD8^+^ epitope alone or both the OVA CD8^+^ and LLO190 CD4^+^ epitopes, as determined by staining with the H-2Kb-SIINFEKL antibody. (**G**) OVA tetramer^+^ T cells in the blood of mice (*n =* 10) injected with Tvax_OVA_ or Tvax_OVA-LLO190_. *P < 0.003 for differences between groups, by Mann-Whitney *U* test.

Prior work in humans, nonhuman primates, and mice showed that T cells bearing transgenes that encode foreign antigens can be immunogenic (22, 23, 25, 26). To examine the mechanisms responsible, we performed retroviral transduction of murine T cells with a construct encoding a murine truncated CD19 (tCD19) surface marker fused to a chicken OVA epitope (OVA_257–264_), which is presented by class I H-2Kb, and to listerolysin O 190-201 (LLO), which is presented by class II I-Ab (Figure 1A and Supplemental Figure 1; supplemental material available online with this article; https://doi.org/10.1172/JCI144195DS1). We administered a single i.v. dose of Tvax or mock-transduced T cells to syngeneic mice and measured T cell responses to OVA and LLO in the blood after vaccination. OVA_257–264_-specific CD8^+^ T cells were detected by tetramer staining 1 week after vaccination in mice receiving Tvax and peaked at 2 weeks (Figure 1, B and C). Tetramer^+^CD8^+^ T cells on day 7 expressed CD44, consistent with effector differentiation, and a fraction of the CD44^+^ T cells expressed CD62L, consistent with the emergence of central memory CD8^+^ T cells (Figure 1D). Four weeks after vaccination, repeat administration of Tvax boosted OVA-specific CD8^+^ T cells to frequencies that were greater than those seen in the initial expansion, indicating that functional memory T cells had been elicited (Figure 1C). We also detected IFN-γ–producing LLO_190–201_-specific CD4^+^ T cells 13 days after a single vaccination with Tvax, albeit at a much lower level than OVA_257–264_-specific CD8^+^ T cells (Figure 1E). Tvax products made from isolated CD8^+^ or CD4^+^ T cell subsets were both capable of priming antigen-specific CD8^+^ T cell responses (Supplemental Figure 2, A and B)

To determine the importance of CD4^+^ T cell help for the CD8^+^ T cell responses elicited with Tvax, we compared responses in mice vaccinated with a construct that contained only OVA_257–264_ linked to tCD19. Although expression of the surface marker CD19 and presentation of the OVA peptide on H-2Kb was modestly higher in the Tvax_OVA_ construct relative to the Tvax_OVA_ _LLO190_ construct (Figure 1F), we observed markedly less priming of OVA-specific CD8^+^ T cells without the presence of a CD4^+^ epitope (Figure 1G). To address the possibility that retroviral transduction could be a source of adjuvant signals, and to determine whether other class II–restricted epitopes could provide CD4^+^ T cell help, we compared vaccination with Tvax transduced with SIINFEKL or SINNFEKL plus LL0190 with Tvax constructed by ex vivo activation of T cells from a donor mouse strain (Act-OVA) with germline expression of full-length OVA. We found that OVA-specific CD8^+^ T cell responses were equivalent with Tvax_OVA_ _LLO190_ and Tvax_OVA_ (Supplemental Figure 2, C and D), indicating that the vaccine was not dependent on adjuvant effects from retroviral transduction and that class II MHC epitopes provided by OVA could function in CD4^+^ T cell help of CD8^+^ T cell responses. Because CD4^+^ T cell help augmented the CD8^+^ T cell response elicited by Tvax, we included the class II MHC–restricted LLO antigen in further experiments with OVA, unless otherwise indicated in the figures.

### Gene-modified T cells traffic efficiently to secondary lymphoid tissue and are taken up by APCs.

**Figure 2:**
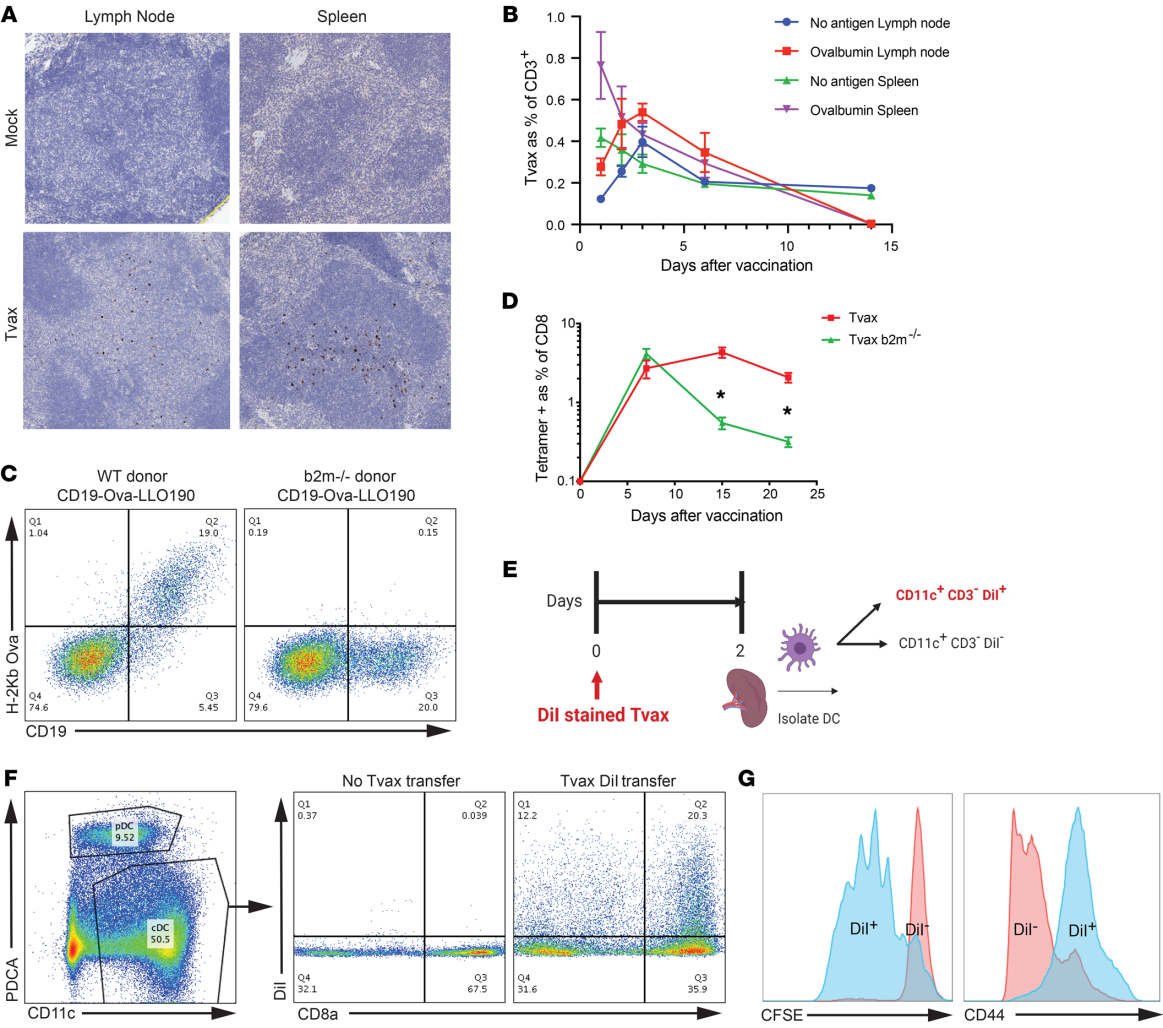
Syngeneic Tvax cells traffic to secondary lymphoid tissues and prime CD8^+^ T cell responses by cross-presentation on host DCs. (**A**) Immunohistochemistry of spleens and lymph nodes from control mice and mice that received Tvax expressing GFP. T cells were injected i.v., and 48 hours later, spleens and lymph nodes were harvested and stained for GFP by immunohistochemistry. Original magnification, ×20. (**B**) Tvax cells were created from WT (no antigen) or full-length OVA-expressing donor mice and labeled with CellTrace Violet (CTV), and 2 × 10^6^ cells were transferred i.v. into mice. The percentage Tvax cells as a fraction of CD3^+^ T cells in spleen and lymph nodes was measured by flow cytometry in mice sacrificed at the indicated time points (*n =* 4 per group). Error bars represent the SEM. (**C**) Staining of tCD19 and SIINFEKL-H-2Kb on T cells from WT and *B2m^–/–^* donor mice transduced with tCD19-OVA-LLO190. (**D**) Tvax cells from WT and *B2m^–/–^* donors were injected into mice, and the percentage of antigen-specific cells in the blood was measured by tetramer staining. (*n =* 10). (**E**) Schematic of dye transfer experiment to determine DC uptake of Tvax cells. (**F**) Splenic DCs were enriched by negative immunomagnetic selection 48 hours after injection of DiI-labeled Tvax cells, and DiI uptake by CD11c^hi^PDCA^–^ cells was measured in CD8a^+^ and CD8a^–^ cell subsets by flow cytometry. (**G**) Naive OT-I CD8^+^ T cells were labeled with CFSE and incubated with CD11c^hi^DiI^+^ and DiI^–^ cells sorted by FACS from mice 48 hours after vaccination. CFSE dilution (left panel) and CD44 expression (right panel) in OT-I T cells was measured 4 days later by flow cytometry. Results are representative of 3 biological replicates from 2 independent experiments. *P < 0.0001, by Mann-Whitney *U* test.

To identify potential sites where immune responses might be elicited in vivo, we evaluated the trafficking of i.v. transferred Tvax cells by modifying T cells with a construct encoding GFP to enable their detection in secondary lymphoid organs (Supplemental Figure 1). GFP^+^ T cells were detected by immunohistochemistry in the T cell zones of lymph nodes and the white pulp of the spleen harvested from mice 48 hours after adoptive transfer (Figure 2A), indicating widespread trafficking of transferred T cells to lymphoid tissue. Expression of CD62L on a majority of Tvax cells prior to injection suggested this as a possible mechanism of lymphoid organ targeting of these cells (Supplemental Figure 2E). Transferred Tvax cells trafficked rapidly to the spleen and peaked in lymph nodes 72 hours after infusion. Tvax cells expressing OVA, but not those that lacked an antigen, were cleared from the spleen and lymph nodes by day 15, a finding consistent with clearance mediated by antigen-specific T cells that were induced by vaccination (Figure 2B).

Immune responses could be induced by Tvax trafficking to lymphoid organs through direct antigen presentation to naive CD8^+^ T cells, or by cross-presentation of antigen after uptake by endogenous APCs. To distinguish these mechanisms, Tvax_OVA-LLO190_ was prepared from β-2 microglobulin–deficient (*B2m^–/–^*) mice that lacked MHC class I. We confirmed the absence of OVA peptide on class I MHC by staining for the OVA peptide–H-2Kb complex (Figure 2C). Infusion of *B2m^–/–^* Tvax resulted in CD8^+^ T cell responses on day 7 that were equivalent to the response elicited by Tvax cells prepared from WT mice, although the responses fell to lower levels by day 14 and day 21 (*P <* 0.0001; Figure 2D). This shows that direct presentation of antigen by Tvax cells is dispensable for the priming of CD8^+^ T cells, but suggests that direct stimulation of primed CD8^+^ T cells may contribute to sustaining a higher response level.

We next asked whether host DCs took up Tvax cells in vivo and cross-presented the antigen expressed by these cells. Tvax_OVA_ cells labeled with the membrane-avid, cell-stable dye DiI (27) were administered to mice. Forty-eight hours later, DCs from dissociated spleens of immunized mice were enriched by negative immunomagnetic selection, and DiI uptake by CD11c^hi^PDCA^–^ cells was measured in CD8a^+^ and CD8a^–^ subsets (Figure 2, E and F). We sorted CD11c^+^ classical DCs that took up DiI and those that did not, and then cocultured them with CFSE-labeled naive OT-I T cells in vitro. DiI^+^CD11c^+^ but not DiI^–^CD11c^+^ DCs induced the proliferation of naive OT-1 T cells and upregulated their expression of CD44 (Figure 2G). Thus, antigen from Tvax cells was transferred to CD11c^+^ DCs in vivo, and these DCs efficiently presented antigen to naive T cells, consistent with a requirement for DCs in priming by a previously reported T cell vaccine (26). It is not clear whether this antigen transfer occurs from dying or live Tvax cells, as has been shown to occur with other cellular vaccines (27).

### Modifications of Tvax that activate innate immunity augment immunogenicity.

**Figure 3:**
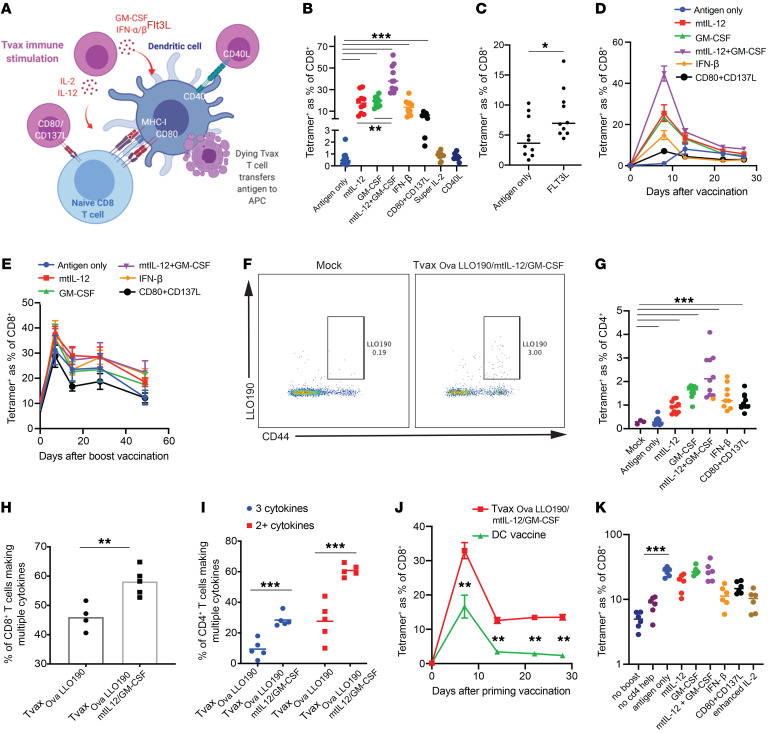
Adjuvant inflammatory signals augment T cell immunity induced by Tvax. (**A**) Schematic of different immunostimulatory Tvax strategies. (**B**–**D**) Tvax cells transduced with antigen only (Tvax_OVA-LLO190_) or with antigen and additional inflammatory signals were injected i.v. into mice. The frequency of OVA-specific CD8^+^ T cell responses in the blood measured by tetramer on day 8 (**B** and **C**) and weekly for 28 days (**D**) is shown for each of the different adjuvants. (**E**) Frequency of OVA-specific T cells in blood after boosting with Tvax_OVA-LLO190_ on day 28. (**F** and **G**) LLO-specific CD4^+^ T cell responses to were measured by tetramer staining on day 7 after vaccination of mice with Tvax and different adjuvants. (**H** and **I**) Splenocytes were incubated with OVA and LLO190 peptides on day 13 after vaccination, and the percentages of polyfunctional CD8^+^ T cells (**H**) and CD4^+^ T cells (**I**) were measured by intracellular cytokine staining for IFN-γ, TNF-α, and IL-2. (**J**) Frequency of OVA-specific CD8^+^ T cells in mice vaccinated with Tvax_OVA–LLO190/mtIL-12/GM-CSF_ or with an identical number of mature DCs pulsed with OVA and LLO190 peptides (*n =* 4 mice per group). (**K**) Mice previously primed with Tvax_OVA–LLO190/mtIL-12/GM-CSF_ were boosted on day 28 with Tvax_OVA-LLO190_ alone or Tvax_OVA-LLO190_ and different additional inflammatory signals. OVA-specific T cell responses were measured in the blood by tetramer staining 7 days after boosting. *P < 0.05, **P < 0.01, and ***P < 0.001, by Mann-Whitney *U* test.

Optimal T cell priming requires engagement of the T cell receptor by peptide/MHC and costimulatory and cytokine signals from APCs, which, in infections, is facilitated by inflammatory signals from innate immune cells (14). We next evaluated whether genetic modification of Tvax to incorporate both antigens and immunostimulatory molecules could increase immunogenicity (Figure 3A). We designed retroviral constructs to express 4 molecules that have a role in the activation and maturation of DCs, including secreted GM-CSF, IFN-β, and FLT3L (28) and membrane-expressed CD40L (ref. 29 and Supplemental Figure 1). We also tested strategies that would directly provide cytokine support or costimulatory signals to activated T cells. We chose a modified form of IL-2 known to stimulate effector T cells independent of CD25 coreceptor expression (30) to potentially augment proliferation of primed T cells, and CD137L and CD80 costimulatory molecules were coexpressed to provide additional direct costimulation to responding T cells (31). In addition, IL-12, which promotes CD8^+^ T cell priming and CD4^+^ Th1 differentiation, was expressed tethered to the Tvax membrane (mtIL-12) to reduce potential systemic toxicity of IL-12 and was incorporated into the Tvax alone or with GM-CSF (refs. 32–36 and Supplemental Figure 1). In each case, we cotransduced the immunostimulatory signal(s) and tCD19-fused antigens with separate retroviral constructs, thus, a limitation was that only a fraction of cells expressed both antigen and putative adjuvant signals (Supplemental Figure 3).

Tvax cells that coexpressed either mtIL-12, IFN-β, GM-CSF, mtIL-12 and GM-CSF, or FLT3L or the combination of CD80 and CD137L produced a markedly greater expansion of OVA-specific CD8^+^ T cells by day 8 following a single vaccination compared with Tvax cells expressing only OVA (antigen only), but Tvax cells coexpressing IL-2 or CD40L did not increase OVA-specific CD8^+^ T cell numbers (Figure 3, B and C). These data indicate that multiple different inflammatory signals can augment Tvax immunity, and some, such as mtIL-12 and GM-CSF together, provide a 25-fold augmentation of the frequency of OVA-specific CD8^+^ T cells in the blood by day 8 over a single dose of Tvax alone, without toxicity. The greater-magnitude responses seen with added inflammatory signals were associated with a higher frequency of CD62L^–^ and CD127^–^ OVA-specific effector T cells on day 8 (Supplemental Figure 4, A–C), with individual mice with the strongest responses showing the highest frequency of CD62L^–^ T cells (Supplemental Figure 4D). Because of the higher number of total OVA-specific T cells elicited by Tvax encoding additional inflammatory signals, however, the absolute number of CD127^+^ and CD62L^+^ OVA-specific T cells on day 22 was also higher, consistent with a greater total recruitment of T cells to the central memory pool (Supplemental Figure 4, E and F). The combination of mtIL-12 and GM-CSF resulted in a remarkable increase in CD8^+^ T cell priming on day 8 (Figure 3B) compared with that observed with either signal alone, and resulted in 20%–60% of all CD8^+^ T cells in the peripheral blood being OVA specific after a single vaccination. In addition, the higher frequency of antigen-specific T cells compared with other Tvax regimens persisted for 28 days (Figure 3D). On day 22, we observed a substantial fraction of CD62L^+^ and CD127^+^ OVA-specific T cells in mice treated with Tvax with and without inflammatory signals (Supplemental Figure 4, G and H), and in each case, these T cells expanded upon rechallenge with antigen on day 28, consistent with the formation of functional T cell memory (Figure 3E). Tvax-induced cells expressed t-bet, and a fraction of these cells expressed KLRG1, but enhancement of Tvax with mtIL-12 and GM-CSF did not increase the expression of t-bet or the percentage of cells expressing KLRG1 (Supplemental Figure 4 I). LLO_190–201_-specific CD4^+^ responses 7 days after vaccination were also enhanced by inflammatory signals, with Tvax_OVA–LLO/mtIL-12/GM-CSF_ eliciting the maximal response (Figure 3, F and G). As was seen with Tvax without inflammatory signals, Tvax prepared from only CD8^+^ or CD4^+^ T cell subsets was equally immunogenic (Supplemental Figure 5A). Addition of mtIL-12 and GM-CSF resulted in a lower frequency of Tvax cells in lymph nodes and spleen, as well as faster clearance of these cells, consistent with a more rapid and robust immune response against the antigen (Supplemental Figure 5B). CD40L-transduced cells were underrepresented among Tvax cells in lymph nodes and spleen (Supplemental Figure 5C), suggesting that reduced trafficking could have contributed to the lack of added immunogenicity seen with CD40L-expressing Tvax cells.

We examined the function of CD4^+^ and CD8^+^ T cells induced by Tvax by restimulating splenic T cells from animals 13 days after vaccination with the OVA_257–264_ and LLO_190–201_ peptides. Staining for intracellular IFN-γ in CD8^+^ and CD4^+^ T cells revealed frequencies of cells similar to those seen following staining for the respective tetramers (Supplemental Figure 6, A–F). When assessed for polyfunctional responses, we found that a higher proportion of CD8^+^ T cells produced both TNF-α and IFN-γ in mice that had been vaccinated with Tvax_OVA–LLO/mtIL-12/GM-CSF_ (Figure 3H). Similarly, a higher fraction of CD4^+^ T cells from mice vaccinated with Tvax_OVA–LLO/mtIL-12/GM-CSF_ were polyfunctional and produced TNF-α, IFN-γ, and IL-2 or 2 of these 3 cytokines (Figure 3I and Supplemental Figure 6, D–G), indicating that Tvax_OVA–LLO/mtIL-12/GM-CSF_ increased the fraction of cells that were polyfunctional compared with Tvax_OVA-LLO190_ alone.

We compared the frequency of T cells elicited by Tvax_OVA–LLO/mtIL-12/GM-CSF_ with that after administration of comparable numbers of peptide-pulsed, activated bone marrow–derived DCs and found higher frequencies of OVA-specific T cells in the blood with Tvax (Figure 3J). These results indicate that the ease with which Tvax cells can be modified with multiple inflammatory signals is an advantage over systemic DC vaccination for eliciting robust T cell responses.

We next evaluated how memory T cell responses elicited by Tvax_OVA–LLO/mtIL-12/GM-CSF_ respond to rechallenge by revaccinating mice on day 28 with Tvax_OVA-LLO_ (antigen only) or Tvax combined with additional inflammatory signals. Tvax cells without adjuvant signals boosted previously induced responses, and this boost was partly dependent on coexpression of a CD4^+^ antigen (Figure 3K). In contrast to the results obtained during priming, we found that boosting was not further enhanced by including mtIL-12 or GM-CSF in the Tvax and trended toward repression by the Tvax containing IL-2, CD80 and CD137L, or IFN-β (Figure 3K). These results indicate that Tvax cells expressing both class I and II–restricted antigens stimulated optimal secondary responses and that the optimal adjuvant signals for priming naive T cells and boosting memory responses are distinct.

### Tvax encoding neoantigens elicits high-frequency T cell responses.

**Figure 4:**
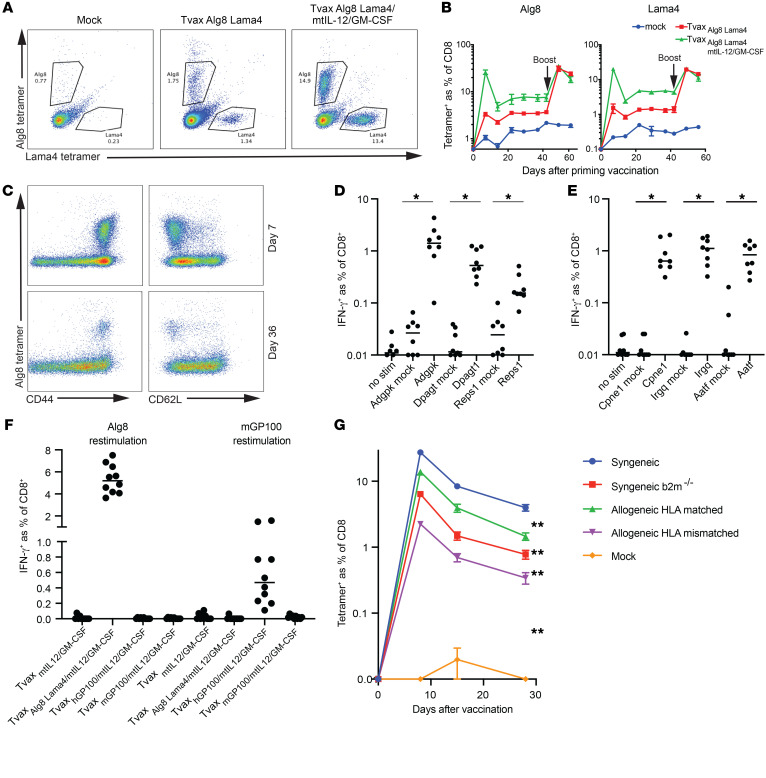
Tvax induces T cell responses to naturally occurring neoantigens. (**A**) Mice were injected with Tvax expressing sequences spanning the Alg8 and Lama4 point mutations previously described in a methylcholanthrene-induced sarcoma, with and without mtIL12 and GM-CSF adjuvants. Alg8- and Lama4-specific T cell responses were measured in the blood on day 7 by tetramer staining. (**B**) Frequency of Alg8- and Lama4-specific T cells in mice over time and after a booster vaccination on day 42 with Tvax expressing Alg8 and Lama4 only. (**C**) Expression of CD44 and CD62L on tetramer^+^ T cells in the blood on day 7 and day 36 following vaccination. (**D** and **E**) T cell responses induced by administration of Tvax_mtIL-12/GM-CSF_ modified to express the MC-38 neoantigens Adgpk, Dpagt, or Reps1 (*n =* 8 mice/group) (**D**), and Cpne1, Irgq, or Aatf (*n =* 8 mice/group) (**E**). Responses were determined by intracellular staining for IFN-γ in CD8^+^ T cells following stimulation of splenocytes pulsed with peptides for each neoantigen. Controls included responses in unstimulated splenocytes and in splenocytes from mice that were not vaccinated. (**F**) Mice were injected with Tvax_mtIL-12/GM-CSF_ expressing either Alg8 and Lama4, murine GP100, or human GP100. Spleens were harvested on day13, splenocytes were restimulated with Alg8 or mouse GP100 peptide, and CD8^+^ T cell responses to each antigen were determined by intracellular staining for IFN-γ. (**G**) Mice (*n =* 10 per group) were injected with Tvax_Alg8-Lama4–LLO190/mtIL-12/GM-CSF_ cells from C57BL/6 (syngeneic), *B2m^–/–^* (syngeneic B2m), S129 (allogeneic HLA-matched), or BALB/c (allogeneic HLA-mismatched) mice, and Alg8-specific T cell responses were measured in the blood by tetramer staining. *P < 0.0001 and **P < 0.01 for comparisons at all time points, by Mann-Whitney *U* test. Error bars represent the SEM. stim, stimulation.

OVA is a foreign antigen that is known to elicit strong CD8^+^ T cell responses in mice. To determine whether Tvax could induce T cell responses to naturally occurring cancer neoantigens, we expressed the Alg8 and Lama4 neoantigens identified in a methylcholanthrene-induced sarcoma (11) fused to the LLO190 epitope and GFP in a retroviral construct. CD8^+^ T cell responses to both Alg8 and Lama4 were induced 7 days after Tvax administration and persisted for 6 weeks. As observed with OVA, the magnitude of the T cell responses to neoantigens was greatly enhanced by Tvax_Alg8–Lama4/GM-CSF/mtIL-12_ compared with Tvax_Alg8-Lama4_ (Figure 4, A and B). Neoantigen-specific T cells uniformly expressed CD44, and a subset of these cells expressed CD62L (Figure 4C), consistent with the induction of effector and central memory T cells. Consistent with this phenotype, a boosting vaccination with Tvax_Alg8-Lama4_ alone resulted in a robust secondary expansion of Alg8- and Lama4-specific T cells (Figure 4B).

Six neoantigens that are predicted to bind to class I MHC were shown to be detectable on the cell surface of MC-38 tumor cells by mass spectrometry (37), but a previous study using standard peptide vaccination with adjuvant showed that only 3 of these were immunogenic, possibly due to a lack of efficient uptake of water-soluble peptide with adjuvant by APCs (38). Remarkably, when encoded in Tvax_mtIL-12/GM-CSF_, all 6 neoantigens elicited T cell responses 14 days after a single vaccination (Figure 4, D and E). Collectively, these data demonstrate that Tvax can induce CD8^+^ T cell responses to a broad variety of neoantigens, including those not amenable to standard peptide vaccination.

To test whether Tvax could break tolerance to potentially immunogenic self-antigens, we used the GP100 antigen, against which autoreactive T cell responses can be induced with peptide vaccination using the homologous human GP100 sequence, but not the murine peptide sequence (39). We created Tvax enhanced with mtIL-12 and GM-CSF expressing either the murine or human GP100 antigen and found that CD8^+^ T cell responses to the murine antigen could be detected only with vaccination with Tvax expressing the human, but not murine, antigen sequence (Figure 4F).

### Allogeneic Tvax is immunogenic and enhanced by HLA matching.

The ability to use allogeneic cells for vaccination would potentially aid in clinical translation to patients. Allogeneic cells differ from syngeneic cells in HLA matching and the ability to directly present antigens to host T cells, and also in other antigenic differences between individuals that could affect clearance. We constructed Tvax_Alg8-Lama4-LLO190_ from syngeneic B6 donors, allogeneic but HLA-matched donors (129S), and allogeneic, HLA-mismatched donors (BALB/c). B6 *B2m^–/–^* donors were used to control for the effect of direct presentation in isolation. mtIL-12 and GM-CSF were included in all preparate Tvax vaccines. We observed a slight decrement of Alg8-specific CD8^+^ T cell responses to the allogeneic matched vaccines and a larger decrement in the response to the allogeneic HLA-mismatched vaccine, with the syngeneic *B2m^–/–^* being intermediate between the 2 allogeneic vaccines (Figure 4G). This indicated that allogeneic Tvax can be immunogenic and that immunogenicity is aided by HLA matching.

### IL-12 augments Tvax immunity through autocrine signaling and production of IFN-γ and can be replaced by a constitutively active IL-12 receptor.

**Figure 5:**
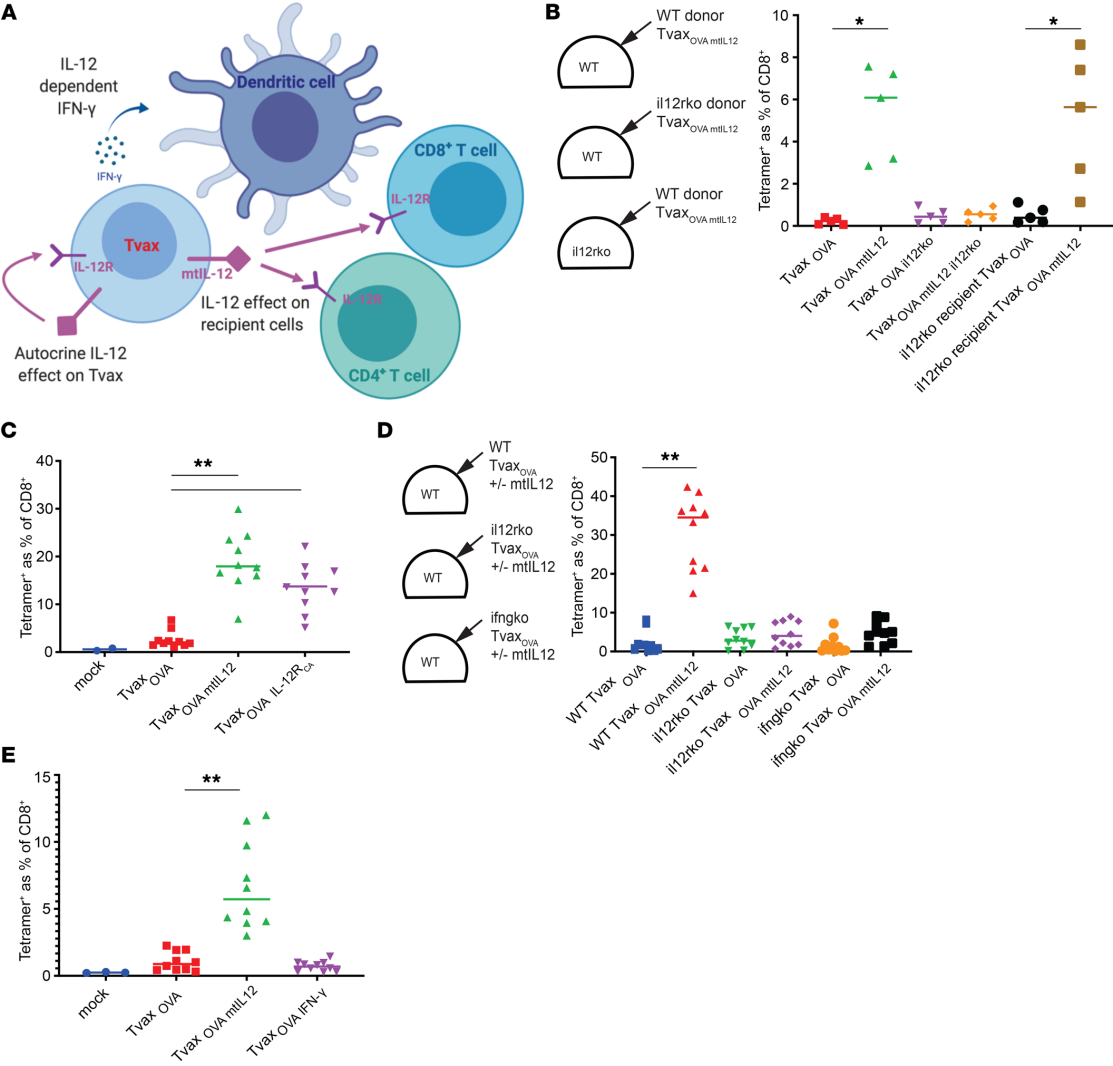
Autocrine effects of mtIL-12 on Tvax cells mediate enhanced immunity of Tvax through IFN-γ. (**A**) Schematic of potential mechanisms whereby IL-12 could augment Tvax priming of CD8^+^ T cell responses. (**B**) Tvax_OVA_ cells were engineered with and without mtIL-12 using T cells from WT mice and from mice lacking the IL-12R (*Il12r*-KO) and then administered to WT or *Il12r*-KO mice (*n =* 5 mice/group). OVA-specific CD8^+^ T cells were measured in the blood 8 days after vaccination by tetramer staining. (**C**) Tvax_OVA_ cells were constructed with either mtIL-12 or IL-12R_CA_ and administered to WT mice. OVA-specific CD8^+^ T cell responses were measured in the blood 8 days following vaccination by tetramer staining. (**D**) Tvax_OVA_ cells from WT or *Il12r*-KO mice or mice lacking IFN-γ (*Ifng*-KO) were administered to mice of the same genotype, and OVA-specific T cell responses were measured by tetramer staining 8 days later (*n =* 10 mice/group). (**E**) Tvax_OVA_, Tvax_OVA/mtIL-12_, or Tvax cells engineered with OVA and constitutively expressing IFN-γ (Tvax_OVA/IFN-γ_) were administered to WT mice, and OVA-specific CD8^+^ T cells were measured in the blood on day 8 by tetramer staining (*n =* 10 mice/group). *P < 0.05 and **P < 0.001, by Mann-Whitney *U* test.

IL-12 could act in several ways to augment CD8^+^ T cell priming, including direct action on activated CD8^+^ T cells (34), stimulation of CD4^+^ Th1 differentiation (32), stimulation of IFN-γ secretion, and an autocrine effect of IL-12 on the Tvax cells (Figure 5A). To better understand the mechanism by which mtIL-12 on Tvax cells enhanced CD8^+^ T cell priming, Tvax_OVA/mtIL-12_ cells alone were prepared from WT donor mice and IL-12 receptor–KO (*Il12r*-KO) donor mice and then administered to WT or *Il12r*-KO mice to assess priming of the OVA-specific T cells. Surprisingly, we found that mtIL-12 on Tvax_WT_ cells enhanced CD8^+^ T cell priming in *Il12r*-KO mice but that mtIL-12 on Tvax_IL-12Rko_ cells did not augment priming in WT mice, consistent with an autocrine effect of mtIL-12 on Tvax cells contributing to improved immunogenicity (Figure 5B).

Signaling downstream of the IL-12 receptor (IL-12R) is activated by heterodimerization of Il12rb1 and Il12rb2 (40). To further examine the role of autocrine IL-12 signaling on Tvax immunogenicity, we expressed a constitutively active IL-12R (IL-12R_CA_) using a method previously described for similar receptors (41). The IL-12R_CA_ consisted of 2 components — the transmembrane and intracellular domains of Il12rb1 fused to a mutant of IL-15 that is unable to bind to CD122/CD132, and the transmembrane and intracellular domains of Il12rb2 fused to the sushi domain of IL-15RA, which binds with high avidity to the mutant IL-15 (Supplemental Figure 1). Expression of the IL-12R_CA_ in Tvax cells led to phosphorylation of STAT4 and induction of IFN-γ secretion (Supplemental Figure 7, A–C) and augmented immunity to almost the same extent as that seen with mtIL-12 (Figure 5C). Thus, an autocrine effect of mtIL-12 on Tvax cells was necessary and sufficient for the enhanced immunity of Tvax expressing mtIL-12.

Autocrine stimulation of IFN-γ secretion in Tvax cells by IL-12 signaling is a candidate mechanism of action for the autocrine effect of mtIL-12, as IFN-γ activates innate immune cells including DCs (42). We tested whether IFN-γ signaling was required for responses elicited by Tvax_mtIL-12_ cells by preparing Tvax_mt-IL12_ cells from mice with a KO of the gene encoding IFN-γ (*Ifng*-KO). The ability of mtIL-12 to augment Tvax immunity was severely reduced by the absence of IFN-γ in Tvax cells (Figure 5D). Constitutive expression of IFN-γ in Tvax cells by retroviral transduction without mtIL-12 did not augment immunity, indicating that IFN-γ alone was not sufficient for this effect (Supplemental Figure 7, D–F and Figure 5E). We also observed the augmentation of immunity by IL-12R_CA_ with the Alg8 neoantigen, indicating that this effect was not specific to the SIINFEKL antigen (Supplemental Figure 7G). This suggests that mtIL-12 augments the immunity of Tvax through both IFN-γ–dependent and IFN-γ–independent pathways and that IL-12 can be functionally replaced by expressing IL-12R_CA_ in Tvax cells, which, for clinical translation, would diminish the potential toxicities of IL-12 expression.

### GM-CSF and mtIL-12 activate host DCs through nonoverlapping mechanisms.

**Figure 6:**
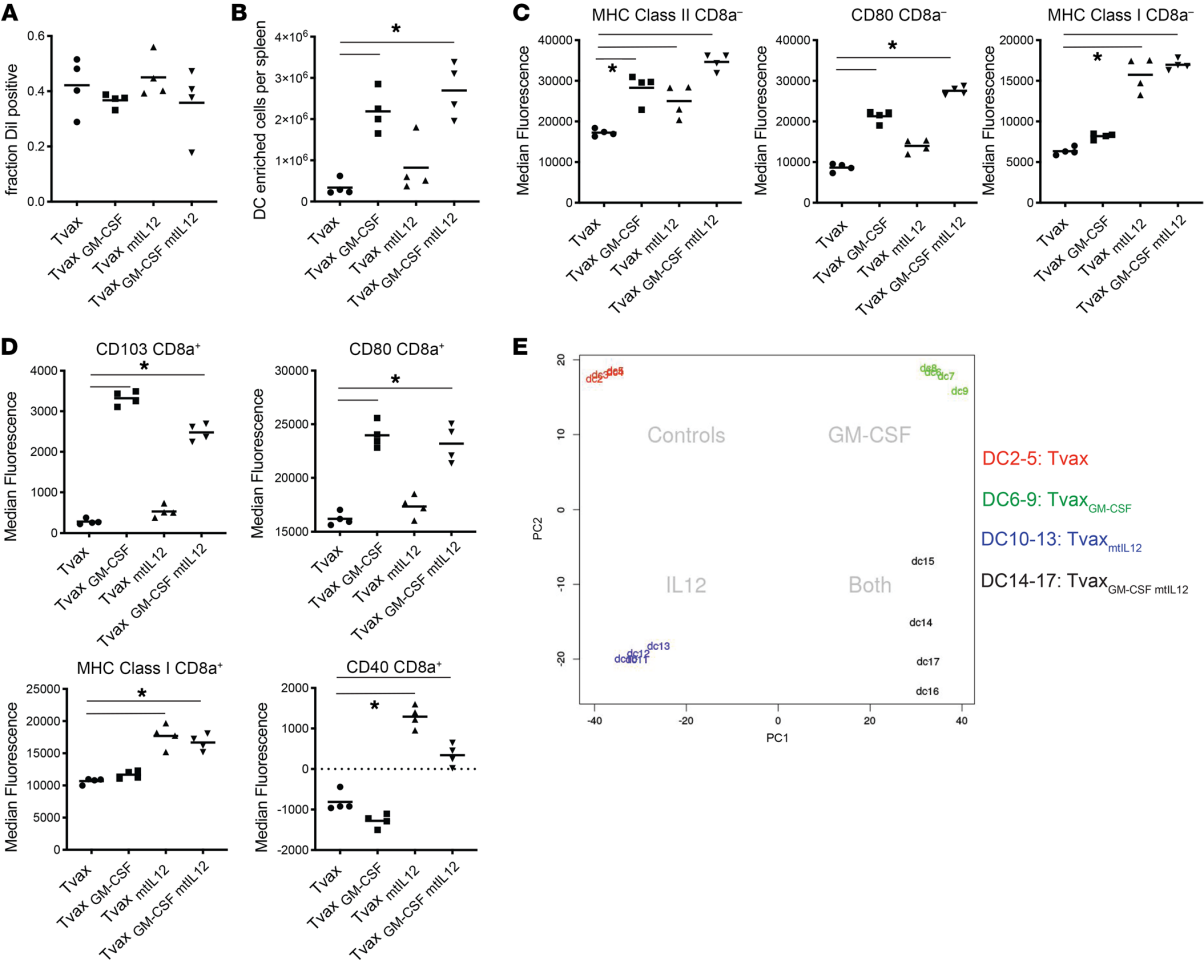
Tvax_mt-IL-12/GM-CSF_ cells stimulate host DCs through complimentary mechanisms. Tvax or Tvax_mt-IL-12/GM-CSF_ cells without antigen were labeled with the lipophilic dye DiI and administered to mice, and splenic DCs were isolated 48 hours after transfer for analysis (*n =* 4 mice/group). (**A**) Fraction of DiI^+^ cells among CD11c^hi^PDCA^–^ cDCs. (**B**) Total number of purified DCs per mouse. (**C**) Expression of MHC class I, MHC class II, and CD80 on CD8a^–^ cDCs was measured by flow cytometry. (**D**) Expression of CD103, CD80, MHC class I, and CD40 on CD8a^+^ cDC was measured by flow cytometry. (**E**) PCA of whole-transcriptome data from DiI^+^ cDCs isolated from individual mice vaccinated with Tvax with mtIL-12, GM-CSF, or both (*n =* 4 mice/group, samples were labeled DC2-17). *P < 0.01, by 1-way ANOVA.

We hypothesized that GM-CSF and mtIL-12 could augment immunogenicity by modulating antigen uptake, numbers, or the activation state of cross-presenting DCs. To investigate the effects of these signals on DCs, Tvax cells without antigens but expressing either GM-CSF or mtIL-12, or a combination of both, were labeled with the lipophilic dye DiI, and splenic DCs that took up antigen were isolated 48 hours after Tvax infusion by negative immunomagnetic selection. We found that expression of mtIL-12, GM-CSF, or both did not result in a larger fraction of CD11c^hi^PDCA^–^ classical DCs (cDCs) staining positive for DiI dye, indicative of Tvax uptake (Figure 6A). However, Tvax cells expressing GM-CSF led to an increase in total DCs isolated from mouse spleens (Figure 6B). GM-CSF and mtIL-12 induced nonoverlapping expression of activation markers, with GM-CSF inducing expression of the costimulatory molecule CD80, mtIL-12 inducing expression of the activation markers CD40 and class I MHC in CD8a^–^ and CD8a^+^ cDCs (Figure 6, C and D), and the combination resulting in upregulation of all of these markers. GM-CSF also led to an increase in CD103 expression among DiI^+^CD8a^+^ cDCs (Figure 6D), consistent with recruitment of DCs to the spleen by GM-CSF (43).

We analyzed transcriptome changes in Dil^+^ DCs after Tvax with or without GM-CSF and mtIL-12. Principle component analysis (PCA) indicated that the transcriptional changes induced in cross-presenting DCs by mtIL-12 and GM-CSF were distinct (Figure 6E), and the combination of the 2 signals induced both sets of changes. Gene set enrichment analysis (GSEA) of the signatures of cross-presenting DCs from Tvax_mtIL-12_–vaccinated mice showed enrichment of a gene set of IFN-γ–activated genes (Supplemental Figure 8A), consistent with a role of IFN-γ produced by Tvax _mtIL12_ in augmenting immunogenicity. GSEA of the signatures of cross-presenting DCs from Tvax_GM-CSF_–vaccinated mice showed enrichment of genes associated with murine CMV (MCMV) infection (Supplemental Figure 8B). Taken together, these observations suggest that GM-CSF and mtIL-12 facilitate the activation of cross-presenting DCs through nonoverlapping mechanisms and that the combination of these adjuvant signals is synergistic for augmentation of T cell responses induced by Tvax.

### Tvax slows tumor growth in transplantable tumor models.

**Figure 7:**
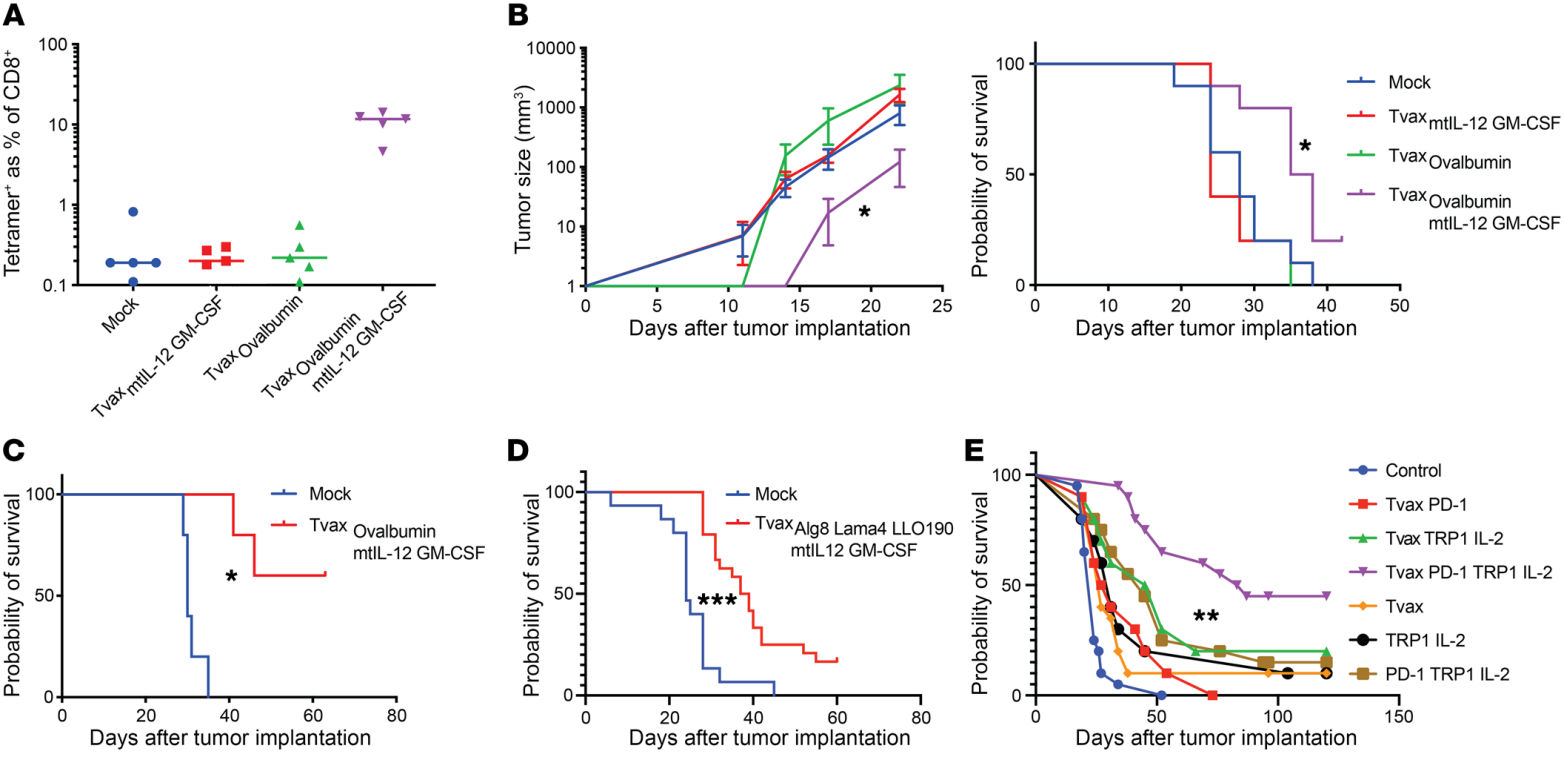
Antitumor effects of Tvax in preclinical models. B16-OVA cells (5 × 10^5^ cells) were injected s.c. into the flank on day 0, and mice were vaccinated (1 × 10^6^ cells) with various Tvax compositions on day 1. (**A**) Frequency of OVA-specific CD8^+^ T cells measured in the blood by tetramer staining on day 7 after Tvax administration (*n =* 5). (**B**) Tumor growth over time measured with calipers (*n =* 10), and survival of tumor-bearing mice administered different Tvax regimens. Error bars represent the SEM. (**C**) B16-OVA cells were injected i.v. into mice on day 0. Mice were vaccinated on day 4, and their survival was measured. (**D**) B16-GFP-Alg8-Lama4-LLO190 tumor cells were injected on day 0. Mice were vaccinated with Tvax_Alg8_ _Lama4_ _LLO/mtIL-12/GM-CSF_ on day 4, and their survival was measured. (**E**) B16-OVA cells were injected into the flanks of mice and on day 11, when tumors were palpable. Then, mice were injected every 7 days with Tvax_mtIL12_ _GM-CSF_ made from either WT donors (control) or OVA-expressing donors (Tvax) in combination with albumin-fused IL-2, an anti–PD-1 antibody, and a tumor-reactive anti-TRP1 antibody (*n =* 10–20/group). *P < 0.05, **P < 0.01, and ***P < 0.0001, by Mann-Whitney *U* test (**B**, left panel) and log-rank test (**B**–**E**).

A key issue is whether Tvax with or without mtIL-12 and GM-CSF could induce T cell responses capable of slowing the growth of established local or metastatic tumors. We tested this in transplantable B16 melanoma, in which full-length OVA was expressed as a model antigen. For these experiments, OVA-expressing Tvax (Tvax_OVA_) cells were created from donor mice that constitutively expressed full-length OVA as a transgene. A single dose of Tvax_OVA_ or Tvax_mtIL-12/GM-CSF_ without OVA did not induce robust OVA-specific T cell responses or slow the growth of B16-OVA tumors. However, Tvax_OVA/mtIL-12/GM-CSF_ induced stronger T cell responses and delayed tumor outgrowth (Figure 7, A and B). In a B16-OVA lung metastasis model, Tvax_OVA/mtIL-12/GM-CSF_ extended survival (Figure 7C). We also tested whether Tvax_Alg8-Lama4-LLO_ could mediate the clearance of B16 tumor cells transduced to express these neoantigens. Although less effective than in the B16-OVA model, Tvax_Alg8–Lama4–LLO/mtIL-12/GM-CSF_ extended the survival of mice inoculated with B16 tumor cells expressing the neoantigens (Figure 7D). Analysis of tumors from mice following euthanasia revealed that a majority of progressing tumors in vaccinated mice had downregulation or loss of the neoantigen transgene, consistent with an effective immune response against antigen^+^ tumor cells (Supplemental Figure 9, A–C).

Treatment of established, vascularized tumors with therapeutic cancer vaccination has been a challenge, given the immune-suppressive tumor microenvironment and acquired T cell dysfunction. Prior work has suggested that vaccination can be combined with inhibition of the programmed cell death 1/programmed death ligand 1 (PD-1/PD-L1) axis, cytokine support, and local activation of the innate immune system locally in the tumor (18). To examine combination therapies that improve Tvax efficacy in established tumors, we combined Tvax_OVA/mtIL-12/GM-CSF_ with long-acting albumin-fused IL-2, an anti–PD-1 antibody, and a tumor-reactive anti-TRP1 antibody in the B16-OVA model, initiating vaccination on day 11 after implantation when tumors were palpable. We used T cells modified with mtIL-12 and GM-CSF with no antigen as a control to account for nonspecific adjuvant effects of the combination. We found that Tvax alone in this model had a modest effect on survival (*P =* 0.029, HR = 0.55) that was not enhanced by PD-1 inhibition alone (*P =* 0.71) but that was greatly enhanced when combined with IL-2, PD-1 inhibition, and an antitumor antibody compared with Tvax alone (*P <* .0001, HR = 0.19; Figure 7E). This effect required both PD-1 inhibition (*P =* 0.018) and the addition of OVA-expressing Tvax (*P =* 0.006). These results indicate that Tvax can mediate therapeutically effective T cell responses in transplantable tumor models expressing OVA or naturally occurring neoantigens, and that optimal therapeutic efficacy in an established tumor model requires combination with other treatment modalities.

### Human Tvax cells present antigen to neoantigen-specific CD4^+^ and CD8^+^ T cells.

**Figure 8:**
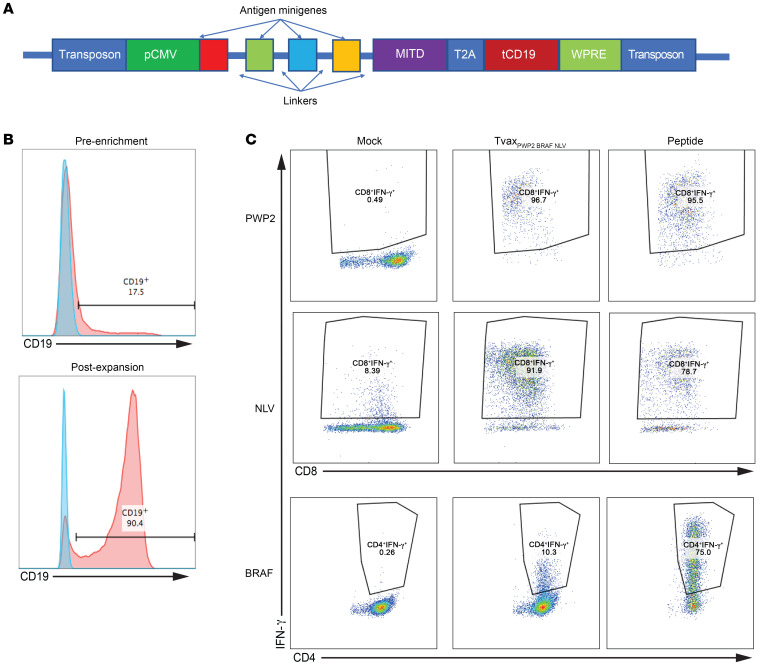
Human T cells present cancer-derived neoantigens. (**A**) Schematic of piggyBac transposon construct to engineer human Tvax. MITD, MHC class I tracking domain; pCMV,cytomegalovirus immediate early promoter; T2A, translational skip sequence from thosea asigna virus; WPRE, woodchuck hepatitis virus posttranscriptional regulatory element. (**B**) Human T cells were transfected with a transposon encoding CMV and tumor neoantigens, and tCD19 and stably transfected cells were measured 7 days later by staining for surface CD19 (top panel). CD19^+^ cells were enriched by immunomagnetic selection and expanded in culture for 14 days followed by CD19 staining (bottom panel) (**C**) T cells from HLA-compatible normal donors were transfected with a transposon containing antigen minigenes and then purified and expanded before use as APCs in vitro. Modified T cells presented the respective antigens to a PWP2-specific CD8^+^ T cell clone derived from a patient with lung adenocarcinoma, a BRAF V600E–specific CD4^+^ T cell clone derived from a patient with melanoma, and CD8^+^ T cells specific for the CMV NLV epitope derived from a healthy donor.

The use of Tvax as a platform for personalized neoantigen vaccination in patients would require the modification of patients’ T cells, preferably using nonviral methods of gene delivery. To evaluate the feasibility of this approach, we used the piggyBac transposon system (44) to express antigens expressed as minigenes fused to an endosomal targeting domain and linked to a surface tCD19 marker by a translational skip sequence (Figure 8A). Human T cells with integrated transposons were then immunomagnetically enriched and expanded more than 1000-fold over 14 days (Figure 8B). We found that expanded Tvax cells did not express PD-L1 (Supplemental Figure 9D). T cells modified with transposons expressing tandem minigenes encoding multiple viral and tumor-associated epitopes connected by linker sequences elicited IFN-γ release from patient-derived CD8^+^ T cells reactive to an MHC class I–restricted viral epitope in CMV, CD8^+^ T cells isolated from a patient with lung adenocarcinoma reactive to a mutation in the PWP2 gene (45), and CD4^+^ T cells isolated from a patient with melanoma specific for the BRAF V600E mutation (ref. 46 and Figure 8C). Thus, human T cells can be readily nonvirally, genetically modified with tandem minigenes expressing neoantigens and function as APCs in vitro, and can be expanded to large numbers for use in therapeutic or preventative vaccines.

## Discussion

Recent human trials have shown that it is feasible to target neoantigens with therapeutic cancer vaccines (8, 9, 19). The demonstration that ICIs alone can induce tumor regression in a subset of patients has led to efforts to develop robust vaccination platforms that induce high-frequency T cell responses and could be used in combination with ICIs. Cell-based vaccines have theoretical advantages in that they enable efficient systemic delivery of antigens to secondary lymphoid organs. We previously found that administration of gene-modified T cells in clinical trials can elicit high-magnitude CD8^+^ and CD4^+^ responses to foreign transgene–encoded antigens, even in highly immunocompromised patients, which suggested that this could be a novel cell-based vaccine platform for cancer (22, 23). Gene-modified T cells that target tumor cells have been adoptively transferred into thousands of patients (47), and these therapies are now approved for a subset of hematologic malignancies. The data presented here show that gene-modified T cells can also be effectively deployed as a vaccine for cancer. In a murine model, we demonstrate that, even in the absence of defined adjuvant signals, Tvax induces robust CD8^+^ and CD4^+^ T cell responses to model antigens and to cancer neoantigens. Transferred Tvax cells trafficked to secondary lymphoid organs, ensuring that antigen was delivered efficiently to a broad repertoire of naive T cells, and the primary mechanism of CD8^+^ T cell priming by Tvax was the transfer of cellular material and cross-presentation by host DCs, which is similar to the mechanism that has been shown for vaccines using DCs (48, 49). Unlike DCs, T cells, including human T cells, can be easily isolated from small amounts of blood, genetically manipulated by viral and nonviral gene delivery, and expanded ex vivo. Indeed, our data show that human cells can be readily modified with tandem minigenes using a nonviral transposon system and can present multiple antigens to neoantigen-specific CD4^+^ and CD8^+^ T cells derived from patients with cancer.

The ease with which T cells could be genetically modified and expanded led us to evaluate the incorporation of additional signals that might enhance their immunogenicity. Expression of Flt3L, IFN-β, GM-CSF, and mtIL-12 or the combination of CD80 and CD137L in Tvax all augmented T cell priming in mice, demonstrating the versatility of this platform for delivering different adjuvant signals. The combination of GM-CSF and mtIL-12 led to the greatest initial T cell expansion, through complimentary effects on the activation of cross-presenting DCs. GM-CSF has been safely used as an adjuvant in tumor cell– and peptide-based vaccines in murine models and in human trials, but IL-12 given systemically has resulted in fatal toxicity (36), raising concerns about the safety of this approach for human translation. One approach to reduce toxicity is to tether IL-12 to the membrane of cells (35), but safety concerns remain, as we observed substantial release of free IL-12 from Tvax cells expressing mtIL-12 (Supplemental Figure 5). Analysis of the mechanism by which mtIL-12 augmented immunogenicity showed that IL-12 functioned in Tvax through an autocrine mechanism, due at least in part to the production of IFN-γ. Based on this understanding, we replaced mtIL-12 in Tvax by incorporating a constitutively active IL-12R, which was as effective as mtIL-12 and should have eliminated the risk of off-target IL-12–related toxicity.

Our data show that Tvax elicited effective antitumor immunity in early treatment in transplantable models of local and systemic cancer. However, therapeutic vaccination has traditionally not been effective as monotherapy in established vascularized tumor models in mice (7), in which effective treatment has required the combination of multiple modalities that support T cell function and engage the innate immune system locally within the tumor (18, 50). Indeed, we show that combinations to augment and support T cell function following Tvax improved efficacy, and we are actively investigating such combination therapies in autochthonous tumor models.

Collectively, our data show that use of autologous T cells can be a remarkably potent vaccine strategy by delivering antigens and adjuvant signals to lymphoid tissues to activate host DCs and promote strong antigen-specific CD8^+^ and CD4^+^ responses to tumor antigens, which can mediate clinical effects in murine models. Tvax could be an effective platform for inducing strong T cell responses in human cancer.

## Methods

### Animals.

C57BL/6 (B6), *B2m^–/–^*, *Ifng^–/–^*, *Il2rb2^–/–^*, *Ifngr^–/–^*, *Il12r^–/–^*, and Act-OVA mice were purchased from The Jackson Laboratory. Hemizygous OT-I mice on a CD45.1 background were generated in-house. Six- to 12-week-old age- and sex-matched mice were used. Mice of the same sex were randomly assigned to the experimental groups or were assigned on the basis of tumor burden, such that mice in all experimental groups had similar average tumor volumes prior to treatment.

### Cell lines.

Lenti-X cells for lentiviral packaging were purchased from Clontech. Plat-E cells for retroviral packaging were purchased from Cell Biolabs. Phoenix-E cells were purchased from Thermo Fisher Scientific. B16 and B16-OVA cells were a gift from Nick Restifo (National Cancer Institute, NIH). B16-OVA, Lenti-X, Phoenix-E, and Plat-E cells were maintained in complete DMEM (Gibco, Thermo Fisher Scientific) with 10% FBS, 2 mM l-glutamine, 100 U/mL penicillin/streptomycin, and 25 mM HEPES. All cells were tested bimonthly for the absence of mycoplasma.

### Cloning of vaccine and adjuvant constructs.

The MP71 retroviral vector was provided by Wolfgang Uckert (Max Delbruck Center, Berlin, Germany). MP71 was digested with EcoRI and NotI and modified using the HiFi DNA Assembly Kit (New England BioLabs [NEB]) with different codon-optimized inserts that were synthesized using GeneArt (Thermo Fisher Scientific). The mtIL-12 construct was created by cloning the membrane-tethered IL-12 from plasmid scIL-12-B7 (35) into MP71. The orientation of transgenes in the plasmid constructs is shown in Supplemental Figure 1, and all plasmids and plasmid sequences are available in Addgene (pMP71-mtIL12; pMP71-CD40L; pMP71-GM-CSF; pMP71-IFN-β; pMP71-tCD19-LLO-SIINFEKL; pMP71-super-IL-2; pMP71-Flt3L; pMP71-tCD19-SIINFEKL; pMP71-IFN-γ; pMP71-mtGFP-llo-lama4-alg8; pMP71-tCD19-llo-mc38tmg; pMP71-mGFP-llo190-mc38minor; pMP71-mIL15-IL12rb1-sushi-il12rb2; pMP71-mGFP-LLO-hPMEL; pMP71-mGFP-LLO-mPMEL; pMP71-mGFP). Antigen minigenes were fused to the C-terminus of tCD19 or EGFP with a 5′ palmitoylation sequence with GGSGG or SSGSS linkers between minigenes. For the CD80/CD137L-expressing viral vector, CD80 and CD137L were cloned from cDNA isolated from B6 splenocytes and cloned into the LZRS retroviral vector at the HindIII site in the orientation CD80 linked by a T2A skip sequence to CD137L. Antigen containing the piggyBac transposon vector was made by cloning a synthesized codon–optimized sequence containing minigenes of 27 amino acids surrounding mutations in BRAF and PWP2 as well as the CMV pp65 NLV epitope linked by a T2A skip sequence to the extracellular and transmembrane domains of human CD19 into the vector PB713B (System Biosciences). Plasmids were verified by restriction digestion and capillary sequencing prior to use, and plasmid (PB713B-pCMV-MITD-NeoMini-T2A-tCD19-WPRE) and full plasmid sequences will be made available in Addgene.

### Generation of Tvax cells.

Retrovirus was produced by transient transfection (Clontech) of Plat-E cells with the indicated MP71 vectors. Viral supernatant was collected 48 hours after transfection and filtered through a 0.45 mm syringe filter (MilliporeSigma). Retrovirus expressing murine CD80 and CD137L was obtained from the supernatant of Phoenix-E cells stably expressing the construct. Five days after transduction, 24-well nontissue culture plates were coated with 12.5 mg/mL RetroNectin (Takara Bio) according to the manufacturer’s protocol, and the plates were loaded with 2 mL filtered virus per well and centrifuged for 2 hours at 3000*g* at 32°C. Murine T cells were enriched from the spleens of B6 mice by negative immunomagnetic selection using the murine T cell isolation kit (STEMCELL Technologies) and stimulated with 1 mg/mL each of plate-bound anti-CD3 and anti-CD28 (clones 145-2C11 and 37.51, respectively, BD Biosciences) for 24 hours in a 37°C, 5% CO_2_ incubator in complete RPMI (RPM1 1640, 10% heat-inactivated FBS, 1 mM sodium pyruvate, 1 mM HEPES, 100 U/mL penicillin/streptomycin, 50 mM β-mercaptoethanol) supplemented with 50U/mL recombinant murine IL-2 (Peprotech). Murine T cells were harvested from anti-CD3/anti-CD28–coated plates and resuspended to 1 × 10^6^ cells/mL in complete RPMI supplemented with 50 U/mL IL-2 and anti-CD3/28 mouse T-Activator Dynabeads (Thermo Fisher Scientific) at a bead/cell ratio of 1:1. Viral supernatant was aspirated from RetroNectin-coated 24-well plates, the wells were rinsed with PBS, and 1 mL (1 × 10^6^) T cells was added to each virus-coated well. The plates were then centrifuged at 800*g* for 30 minutes at 32°C and returned to 37°C, 5% CO_2_ incubators. Four days after transduction, the magnetic beads were removed, and T cell transduction was measured by flow cytometry. The number of Tvax cells injected into mice in the various treatment groups was normalized on the basis of the expression on tCD19 cells, such that each mouse received the same dose of antigen^+^ Tvax cells. Cotransduction of the antigen-encoding virus with 2 or more constructs was performed using 0.5 mL filtered virus from each of the different vectors that was mixed with 1 mL antigen-containing virus, keeping the total volume of virus per well at 2 mL. Transductions were performed in RetroNectin-coated wells of a 24-well plate. Six- to 8-week-old male mice were injected i.v. (retro-orbitally) with 1 × 10^6^ antigen^+^ Tvax cells.

### Generation of the DC vaccine.

Bone marrow cells from 6-week-old C57BL/6 mice (The Jackson Laboratory) were extracted from leg bones with a 25 gauge × 5/8 needle (Terumo). Red blood cells were eliminated by treatment with red blood cell lysis buffer (MilliporeSigma) at room temperature, following the manufacturer’s protocol. After centrifugation, the cells were washed 3 times with RPMI 1640 (Gibco, Thermo Fisher Scientific). The cells were then resuspended and cultured for 10 days in RPMI 1640 containing 1 mM sodium pyruvate, 50 μM thioglycerol, 25 μg/mL penicillin streptomycin (Gibco, Thermo Fisher Scientific), 10% FCS (Gibco, Life Technologies, Thermo Fisher Scientific), and 50 ng/mL mouse GM-CSF (Peprotech), in a humidified incubator at 37°C with 5% CO_2_ (51). Prior to vaccination, DCs were activated with 1 μg/mL LPS (MilliporeSigma) overnight and incubated with 10 μg/mL SIINFEKL and 10 μg/mL LLO190 peptides for 2 hours prior to washing and i.v. transfer into mice.

### Preparation of tissues.

Cell suspensions were prepared from murine spleen and peripheral lymph nodes by tissue disruption with glass slides, filtering through a 40 μm filter, and lysing with ACK lysis buffer (Gibco, Thermo Fisher Scientific). Murine peripheral blood was collected by retro-orbital venipuncture into EDTA FACS tubes followed by 2 rounds of lysis with ACK lysis buffer (Gibco, Thermo Fisher Scientific). Organs for immunohistochemical analysis were fixed in buffered formalin.

### Flow cytometry.

Cells were stained using the LIVE/Dead Fixable Aqua Dead Cell Stain Kit (Invitrogen, Thermo Fisher Scientific) according to the manufacturer’s protocol. For surface staining, cells were incubated at 37°C for 60 minutes in staining buffer (PBS, 2% FBS) with the following directly conjugated antibodies for murine proteins (from BioLegend unless otherwise specified) for stains with tetramers, and at 4°C for 30 minutes for stains without tetramers: CD8a-PE-Cy7 (clone 53–6.7); CD127-PE (clone A7R34); CD44-V500 (BD Horizon, clone IM7); CD62L-APC-Cy7 (clone MEL-14); PD-1 FITC (clone 29F.1A12); KLRG1 PerCP Cy5.5 (2F1/KLRG1); CD19-BV421 (clone 6D5); H2kb-SIINFEKL PE (clone 25-D1.16); IL-12p40-APC (clone C15.6); HA tag A488 (clone 16B12); CD11c e780 (Invitrogen, Thermo Fisher Scientific, clone N418); CD80-APC (clone 16-10A1); H-2Kb–BV605 (clone AF6-88.5); CD40–Pacific blue (clone 3/23); PDCA-1 FITC (clone 927); CD3 PerCP-Cy5.5 (clone 145-2C11); and anti-DYKDDDDK tag PE (clone L5).

Tetramers for SIINFEKL, Alg8, and Lama4 antigens were made in-house at the Fred Hutchinson Immune Monitoring Core and stained at a dilution of 1:250 with anti–mouse CD8a (clone 53-6.7, BioLegend) at a dilution of 1:250 and with other antibodies at 1:100. Disulfide-locked LLO190 1-A(b) tetramer was obtained from the tetramer core facility of the NIH and was stained in flow buffer at a concentration of 1:50 for 60 minutes with other surface antibodies.

For intracellular cytokine staining following restimulation, 1.5 × 10^6^ splenocyte were stimulated with 1 μg/mL SIINFEKL and NEKYAQAYPNVS peptides (AnaSpec) or with minimal peptides for published MC-38 neoantigen sequences (90% pure, ELIM Biopharm) in 0.2 mL complete RPMI in 96-well U-bottomed plates (Costar) at 37°C and 5% CO_2_ for 5–8 hours in the presence of GolgiPlug (BD) before staining for flow cytometry, as described above. The following antibodies (all from Thermo Fisher Scientific) were used: CD8-e780 (Thermo Fisher Scientific); CD44-V500 (clone IM7; BD Biosciences); and CD4–Pacific blue (clone RM4-4), IFN-γ APC (clone XMG1.2), TNF-α FITC (clone MP6-XT22), and IL-2 PE-Cy7 (clone JES6-5H4) (all from BioLegend). For functional analysis of T cells from lung, single-cell suspensions from lungs were incubated with CD45.1 splenocytes (1.5 × 10^6^ cells per well) to serve as APCs and treated as described above with addition of CD45.2 BV510 (clone 104) and CD45.1 PerCP Cy5.5 (clone A20) (all from BioLegend) with gating out of CD45.1 splenocytes during analysis.

Staining of cells for phosphorylated STAT4 was accomplished by fixation with Phosflow Fix Buffer (BD Biosciences) and permeabilization with Phosflow Perm Buffer III (BD), followed by staining with anti–STAT4-pY693-PE (BD) and anti–MYC Alexa Fluor 488 (Thermo Fisher Scientific), both at dilutions of 20:1 following the manufacturer’s instructions (https://www.bdbiosciences.com/en-eu/mouse-splenocytes).

Data were acquired on a FACSCanto 2 or Symphony flow cytometer (BD Biosciences) and analyzed with FlowJo software (Tree Star).

### DiI staining and isolation of DCs.

To evaluate the migration and uptake of Tvax, the Tvax cells (1 × 10^6^/mL) were washed with warmed RPMI and stained with the carbocyanine dye DiI (Vybrant DiI, Thermo Fisher Scientific) at a concentration dilution of 1:200 in warm RPMI for 20 minutes with rotation. Cells were washed 3 times with serum containing medium prior to injection. Spleens from injected mice were harvested 48 hours after cell injection, and DCs were enriched using the Mouse Dendritic Cell Isolation Kit (STEMCELL Technologies) following the manufacturer’s instructions. PDCA^–^CD11c^hi^CD3^–^DiI^+^ and PDCA^–^CD11c^hi^CD3^–^DiI^–^ cDCs were sorted using a FACSAria (BD Biosciences). For in vitro stimulation experiments, 10,000 sorted DCs were irradiated (3500 cGy) and incubated with 10^5^ naive OT-I T cells that had been isolated from the spleens of OT-I mice using the mouse CD8 Purification Kit (STEMCELL Technologies) and stained with CFSE for 10 minutes in PBS. Following 4 days of coculturing, T cells were analyzed for CFSE and CD44 expression by flow cytometry. For RNA-Seq experiments, 10^5^ sorted PDCA^–^CD11c^hi^CD3^–^DiI^+^ cells were used to isolate RNA using the RNeasy Micro Kit (QIAGEN) to create cDNA libraries for sequencing.

### Tumor models.

For the B16 melanoma flank tumor model, 5 × 10^5^ B16-OVA tumor cells were injected into the flanks of 5- to 7-week-old male C57BL/6J mice, and tumors were measured with calipers twice weekly.

B16-Alg8-Lama4-LLO190 tumor cells were created by transducing B16 cells with a retrovirus made from MP71-EGFP-Alg8-Lama4-LLO190, and GFP^+^ cells were enriched by FACS. For the i.v. lung metastatic model, 5 × 10^5^ B16-OVA or B16-Alg8-Lama4-LLO190 cells were injected into the tail vein of 5- to 7-week-old male B6 mice 4 days prior to vaccination. For the established flank model, 200,000 tumor cells were injected into the flank 11 days prior to vaccination and initiation of the combination therapies. Murine albumin–fused murine IL-2 was synthesized at the FHCRC protein core facility and designed as previously described and was injected intraperitoneally at 30 μg/week (18). Anti–PD-1 (clone RMP1-14, 200 μg/week, intraperitoneally) and anti-TRP1 (clone TA99, 100 μg/week, intraperitoneally) were obtained from Bio X Cell.

### Immunohistochemistry.

Formalin-fixed, paraffin-embedded tissues were sectioned at 4 μm onto positively charged slides and baked for 1 hour at 60°C. The slides were then dewaxed and stained on a Leica BOND Rx Autostainer using Leica BOND reagents for dewaxing (BOND Dewax Solution, Leica). Antigen retrieval was performed at 100°C for 35 minutes using Leica Epitope Retrieval Solution 2. Endogenous peroxidase was blocked with 3% H_2_O_2_ for 5 minutes, followed by protein blocking with TCT buffer (0.05 M Tris, 0.15 M NaCl, 0.25% casein, 0.1% Tween-20, pH 7.6 ± 0.1) for 10 minutes. Primary rabbit anti-GFP at 1:1000 (Invitrogen, Thermo Fisher Scientific, A11122) was applied for 60 minutes, followed by Leica Rabbit-HRP polymer for 12 minutes (Leica DS9800), and staining was visualized using BOND Polymer Refine Detection DAB for 10 minutes (Leica DS9800). The sections were counterstained with hematoxylin for 4 minutes and then coverslipped. Rat anti-CD8a (clone 4SM15, catalog 14-0808-82, eBioscience) was used at 1:500 for 60 minutes, followed by ImmPRESS goat anti–rat IgG HRP polymer (Vector MP-7444) for 60 minutes, and both antibodies were blocked and detected as described above. The percentage of CD8a^+^ cells within tumors was quantified using HALO software (Indica Labs).

### RNA-Seq.

Samples were barcoded, pooled, and sequenced on the Illumina HiSeq 2500 in “Rapid Run” mode using a paired-end 50 bp strategy. Image analysis and base calling were performed with Real-Time Analysis software, version 1.18 (Illumina), followed by demultiplexing of indexed reads and generation of FASTQ files with Illumina’s bcl2fastq Conversion Software, version 1.8.4 (http://support.illumina.com/downloads/bcl2fastq_conversion_software_184.html).

Read pairs passing standard Illumina quality filters were mapped to the GRCm38/mm10 mouse reference genome with the STAR2 version 2.7.3a aligner in 2-pass mode (52). Gene-level counts were generated from the resulting BAM alignment files with the FeatureCounts program from Subread, version 1.6.4 (53) using Ensembl release 93 annotations for protein-coding genes and long, intergenic noncoding RNAs (lincRNAs).

Differential expression analysis was conducted with edgeR, version 3.28.1 (54) in the R 3.6.2 programming environment. Raw per-gene counts for each sample were merged and filtered to remove low-abundance genes in each comparison. The trimmed mean of *M* values (TMM) method was used to compute normalization factors for each sample, and likelihood ratio tests for differential expression were conducted with the edgeR functions glmFit() and glmLRT().

To characterize differentially expressed genes in each comparison, we applied 2 complementary gene set test methods: “camera” from the edgeR package (55) and “fgsea”(56). The fgsea 1.12.0 method is a fast, preranked GSEA. On the basis of the edgeR results, the genes were ranked by significance and the direction of up- or downregulation (sign[logFC] * -log10[PValue]) and tested with fgseaMultilevel. The fgsea output included, for each gene set tested, enrichment scores, *P* values adjusted for multiple testing with the Benjamini and Hochberg method, and a list of “leading-edge” genes that contributed most to the enrichment score of that gene set.

Camera provides an alternative method to determine whether a gene set is enriched for differentially expressed genes compared with genes not in the set. Camera does not require explicit preranking of genes. It instead operates directly on a DGEList object generated by edgeR, making use of the underlying generalized linear model framework, and adjusts for an inter-gene correlation estimated from the expression data.

These gene set tests were applied to the “C7 Immunologic” gene set collection from MSigDB 7.

To match mouse annotations used for quantitation and differential expression, human gene symbols in the “C7 Immunologic” gene sets from MSigDB were converted to mouse symbols using Ensembl Biomart ortholog lists, and our GSE60501 gene sets (NCBI Gene Expression Omnibus [GEO] database) were added to form the final collection of 4882 gene sets evaluated with the fgsea and camera methods.

### Transposon modification of human T cells.

Human T cells were isolated from cryopreserved PBMCs using the Human T Cell Isolation Kit (STEMCELL Technologies) and cultured overnight with 5 ng/mL human IL-15 (Peprotech) in RPMI media with l-glutamine (300 mg/L) and HEPES (25 mmol/L; both from Gibco, Thermo Fisher Scientific) supplemented with 10% human serum (heat-inactivated; produced in-house from pooled healthy donors), 50 μmol/L β-mercaptoethanol, penicillin (100 U/mL), streptomycin (100 U/mL), and 4 mmol/L l-glutamine). T cells were then electroporated with 5 μg transposon plasmid DNA and 5 μg enhanced piggyBac transposase DNA (PB200A-1, System Biosciences) using the human unstimulated T Cell Nucleofection Kit (Lonza) and program U-014 on a nucleofector 2b (Lonza), followed by stimulation with Dynabeads (Thermo Fisher Scientific) at a 3:1 bead/cell ratio in RPMI media with l-glutamine and HEPES (Gibco, Thermo Fisher Scientific) supplemented with 10% human serum (produced in-house), 50 M β-mercaptoethanol, penicillin and streptomycin, and 4 mM l-glutamine (termed CTL media) with IL-15 (5 ng/mL) for 1 week. After 1 week, T cells expressing the tCD19 transduction marker were enriched using CD19 Magnetic Beads (Milltenyi Biotec) following the manufacturer’s instructions. T cells were further expanded using a rapid-expansion protocol as described previously (57).

### Statistics.

Experimental data was analyzed using GraphPad Prism (GraphPad Software). Frequencies of T cells following priming vaccination were not normally distributed and were compared using the Mann-Whitney *U* test with Bonferroni’s correction for multiple comparisons. T cell and DC phenotypes were compared by 1-way ANOVA. Survival curves were compared by log-rank test.

### Study approval.

All mice were housed and bred at the FHCRC, and experiments were performed in accordance within the guidelines and approval of the FHCRC IACUC. Human materials used in this study were obtained from patients by written informed consent following a protocol approved by the FHCRC IRB.

## Author contributions

JRV, NS, SS, JLS, BJ, and SRR designed experiments. JRV, NS, JLS, BJ, SMS, MS, SYG, and SEJ performed experiments. JRV, SS, MF, and SRR analyzed the data. JRV, NS, and SRR wrote the manuscript.

## Supplementary Material

Supplemental data

## Version 1 08/16/2021

Electronic publication
