# Supplementary material for: A therapeutic cancer vaccine delivers antigens and adjuvants to lymphoid tissues using genetically modified T cells
Source: J Clin Invest. 2021 Aug 16;131(16):e144195. doi: 10.1172/JCI144195 (PMC8363286; doi:10.1172/JCI144195)
Supplement: Supplemental data [file jci-131-144195-s137.pdf]

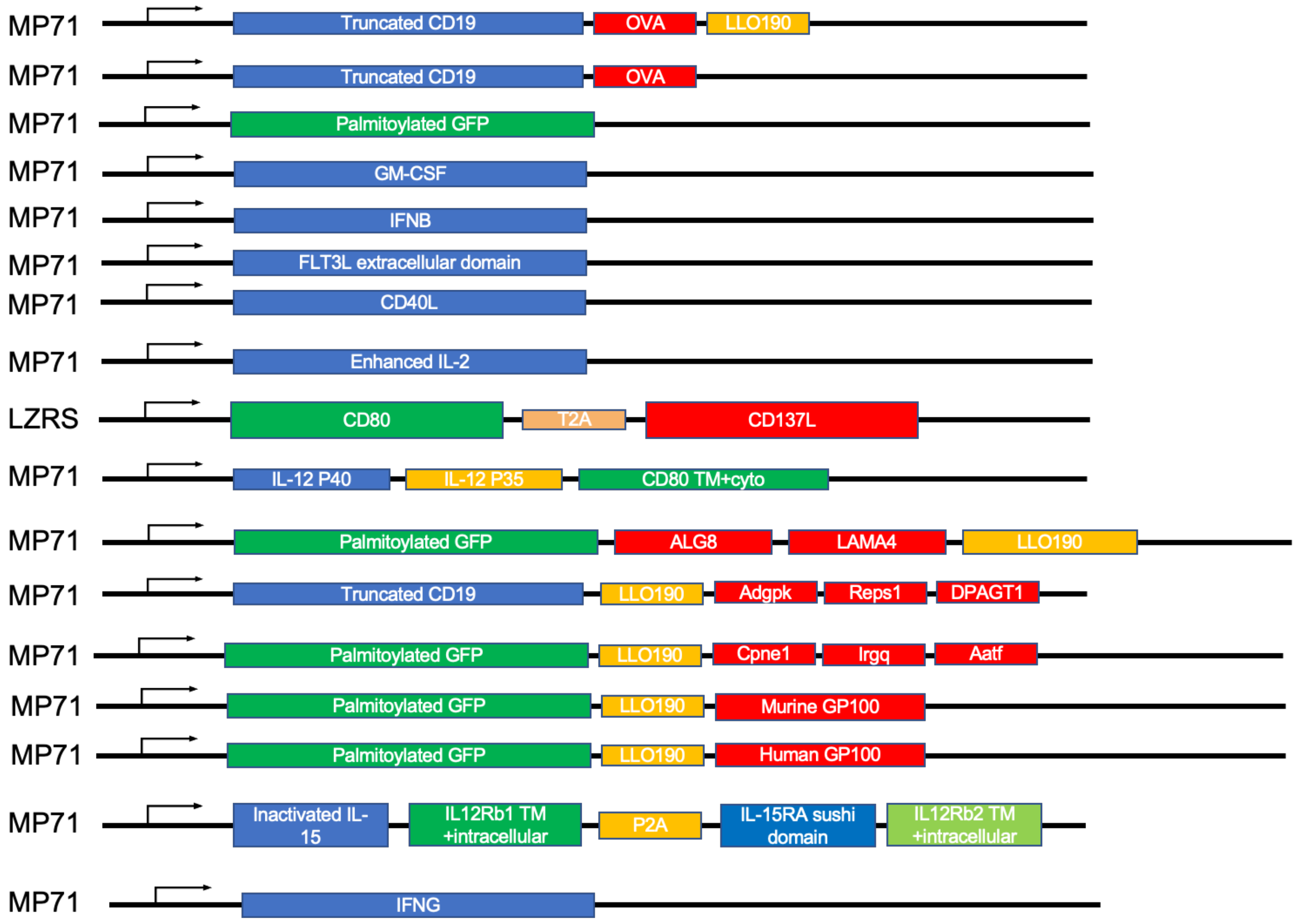

Fig. S1. Schematic of retroviral constructs encoding antigens and adjuvants used in this study

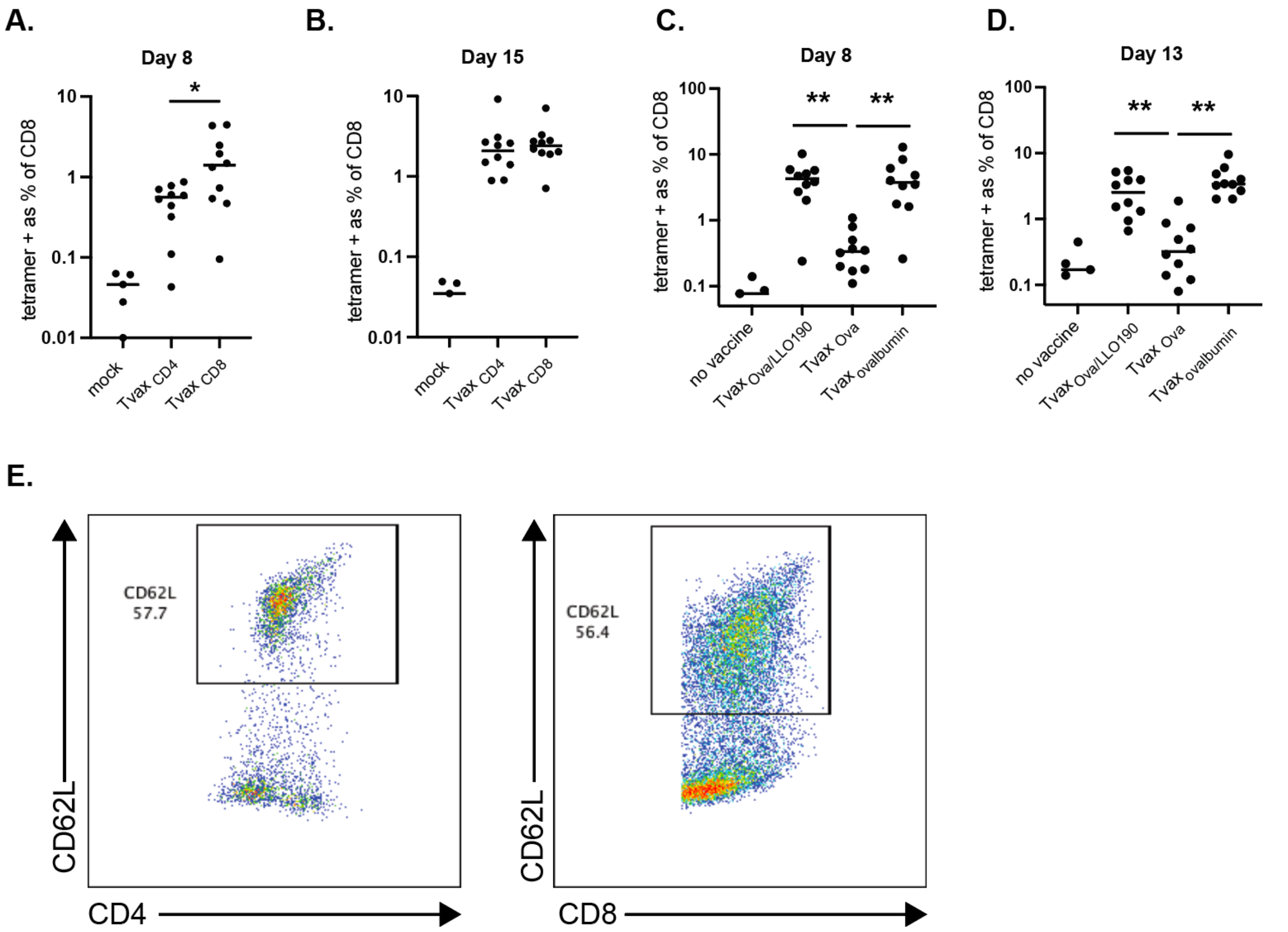

Fig. S2. Immunogenicity of different Tvax preparations.
A-B. Tvax prepared by transduction of donor CD8 or CD4 T cells with Ova and LLO190 were injected intravenously into recipient mice and the frequency of Ova specific CD8+ T cells were measured by tetramer at day 8 (A) and day 15 (B). C-D. Tvax expressing Ova were prepared by mock transduction of T cell isolated from a donor constitutively expressing full length ovalbumin ($Tvax_{Ovalbumin}$) or by transducing wildtype donor T cells with expressing SIINFEKL alone ($Tvax_{Ova}$) or Ova in combination with LLO190 ($Tvax_{Ova\ LLO}$) and Ova specific CD8+ T cell responses were measured at day 8 and day 13 in the peripheral blood by tetramer staining. E. Tvax cells were transduced and prior to infusion CD4+ and CD8+ Tvax cells were measured for surface expression of CD62L by flow cytometry. *p=0.036 **p<0.001

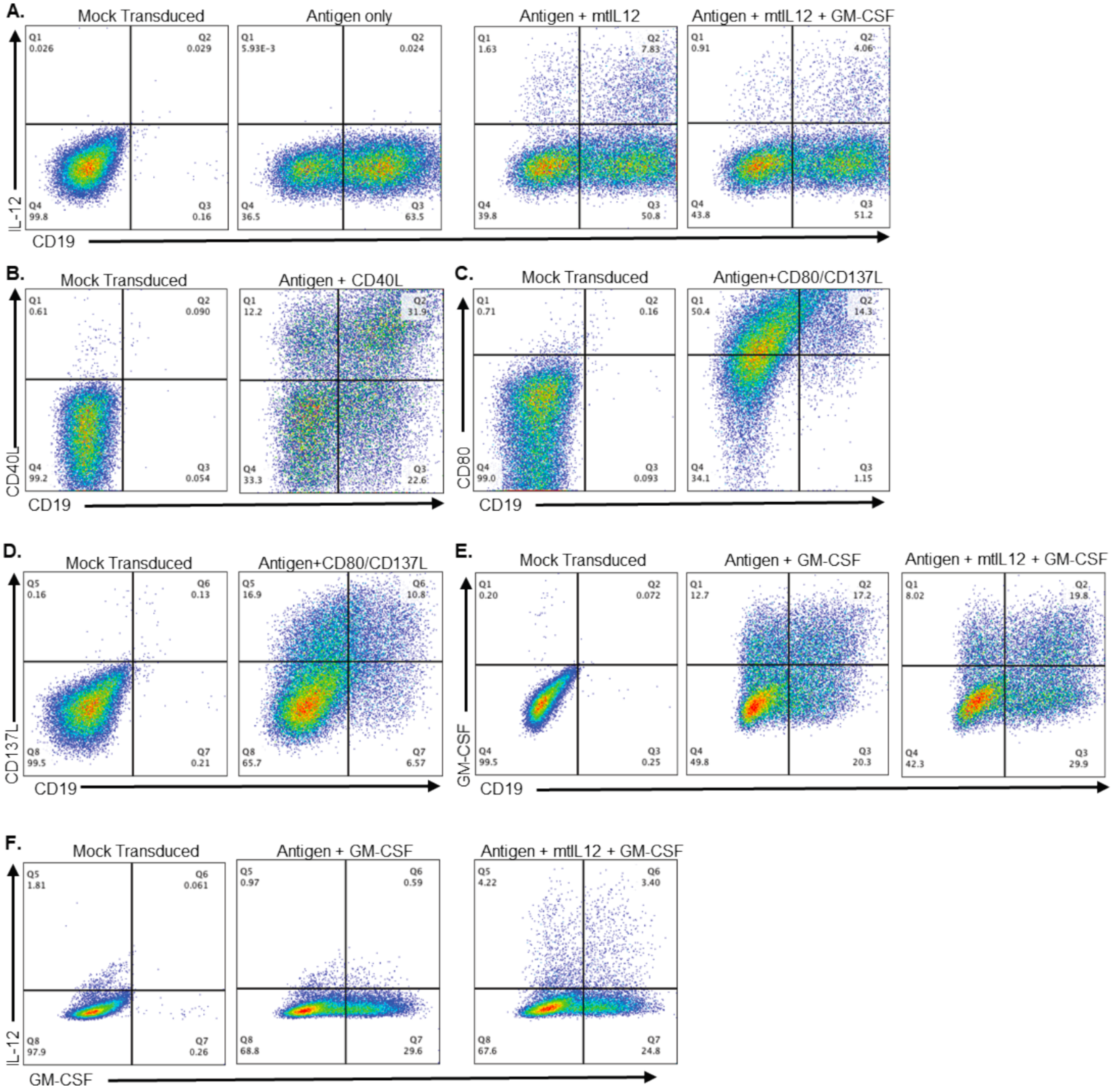

Fig. S3. Co-expression of antigen and adjuvant molecules in Tvax by retroviral transduction.
T cells were isolated from mice and transduced with combinations of retroviruses and stained with surface markers for the transduced antigen (fused to a tCD19) and antibodies to A. IL-12, B. CD40L, C-D. CD80 and CD137L. E-F. T cells transduced with GM-CSF alone or with mt IL-12 were incubated with brefeldin A to block secretion, fixed, permeabilized and stained with antibodies to GM-CSF, CD19 and IL-12.

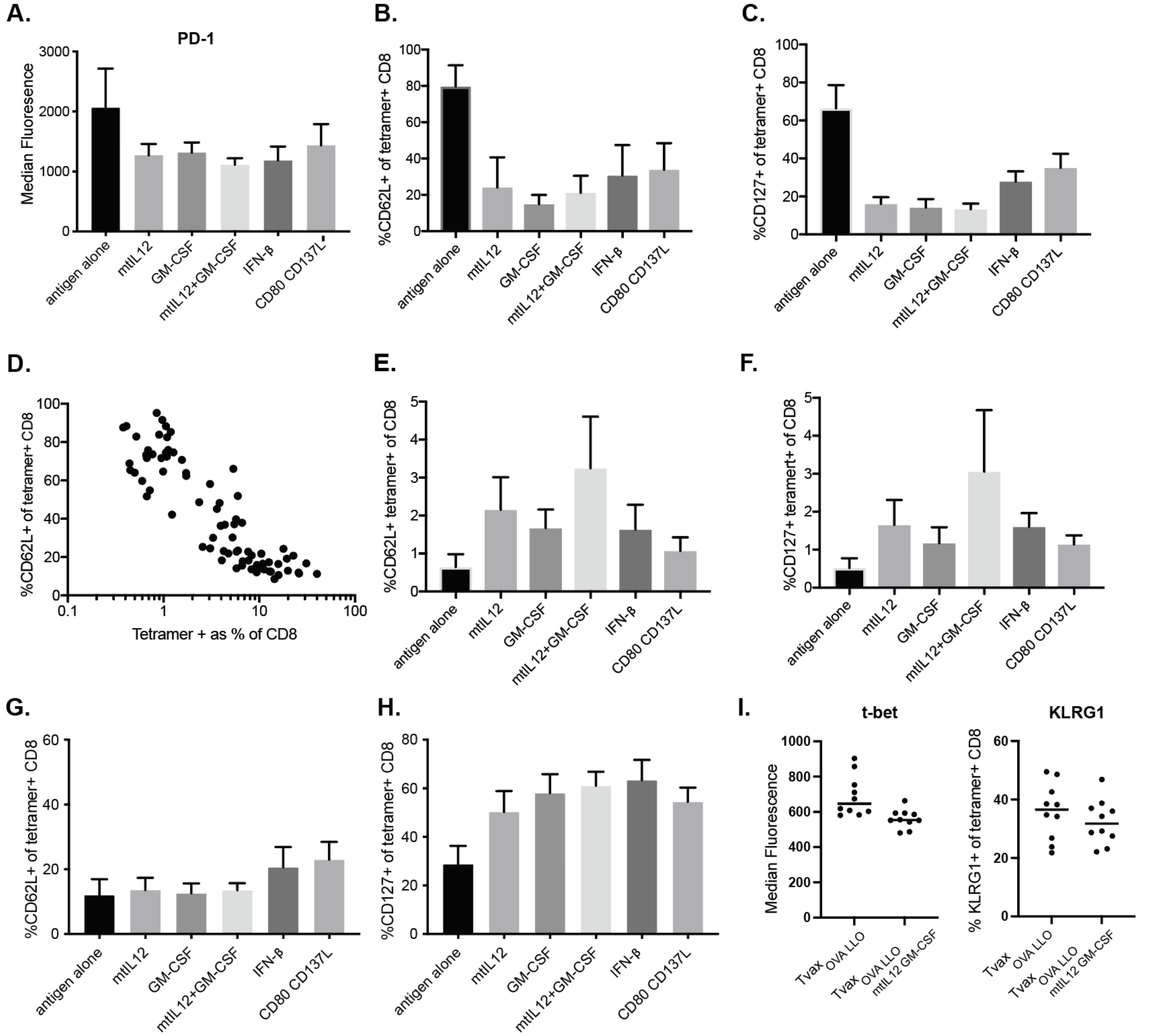

Fig. S4. Phenotype of CD8⁺ T cells induced by Tvax with different adjuvants.
A. The median fluorescence intensity of PD-1 expression on SIINFEKL/H-2Kb tetramer$^+$ T cells on day 8 following vaccination with each of the Tvax regimens. B-C. The percentage of CD62L$^+$ or CD127$^+$ cells as a fraction of tetramer$^+$ CD8 T cells that on day 8 following vaccination with each of the Tvax regimens (n=10 per group). D. The percentage of CD62L$^+$ tetramer$^+$ CD8$^+$ T cells plotted against the log of the percentage of tetramer$^+$ CD8$^+$ T cells in all groups (n=80). E-H. The percentage of CD62L$^+$ tetramer$^+$ or CD127$^+$ tetramer$^+$ cells as a fraction of CD8+ T cells (E,F) or the percentage of CD62L$^+$ tetramer$^+$ or CD127$^+$ tetramer$^+$ cells as a fraction of tetramer$^+$ cells (G,H) at day 22 following vaccination (n=10 per group). I. 500 CD8+ OT-I transgenic T cells were transferred intravenously into donor mice followed by vaccination the following day with the indicated Tvax. KLRG1 expression and t-bet expression were measured at day 8 following vaccination in OT-I cells (n=10 per group).

A.

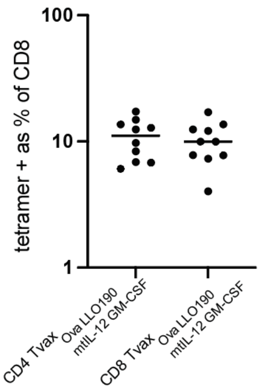

B.

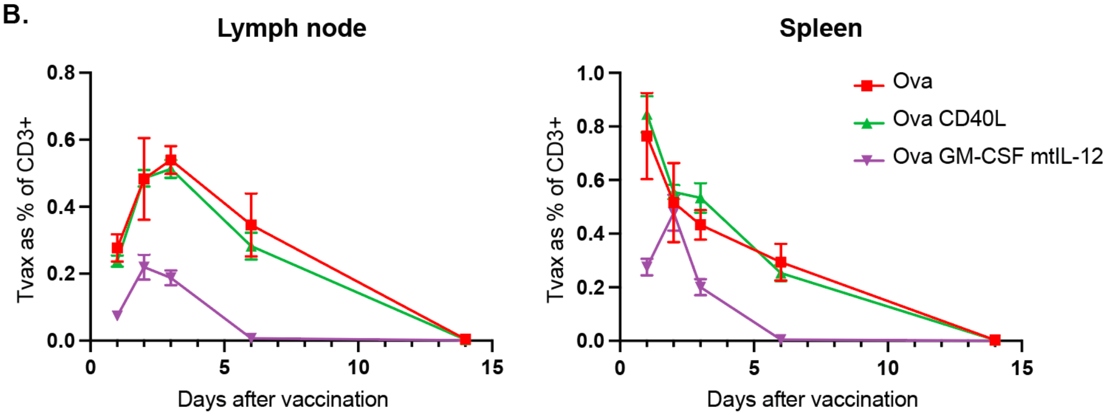

C.

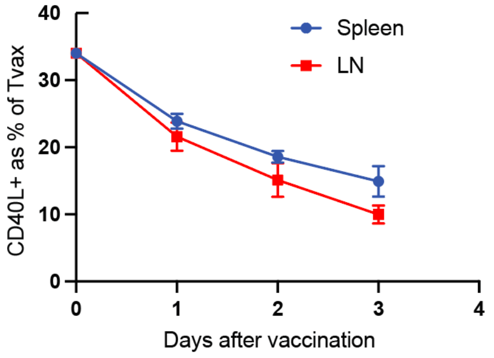

Fig. S5. Trafficking of Tvax with adjuvant signals.

A. Tvax was constructed with from CD8 or CD4 T cells from wildtype donors and transduced with Ova LLO190, mtIL-12 and GM-CSF and the frequency of Ova specific CD8+ T cells in the peripheral blood was measured on day 8 after vaccination by tetramer staining. B-C. Tvax was constructed from T cells from a donor constitutively expressing full length ovalbumin, and mock transduced (Ova) transduced with CD40L or transduced with mtIL-12 and GM-CSF. 10e6 Tvax cells were labelled with cell trace violet (CTV) and transferred into recipient mice with spleen and lymph node were harvested at the indicated times. The frequency of Tvax cells as a fraction of total CD3+ cells was measured by CTV fluorescence (B) and the frequency of Tvax cells expressing CD40L was assessed by flow cytometry (C), with time 0 reflecting the frequency of the infused Tvax.

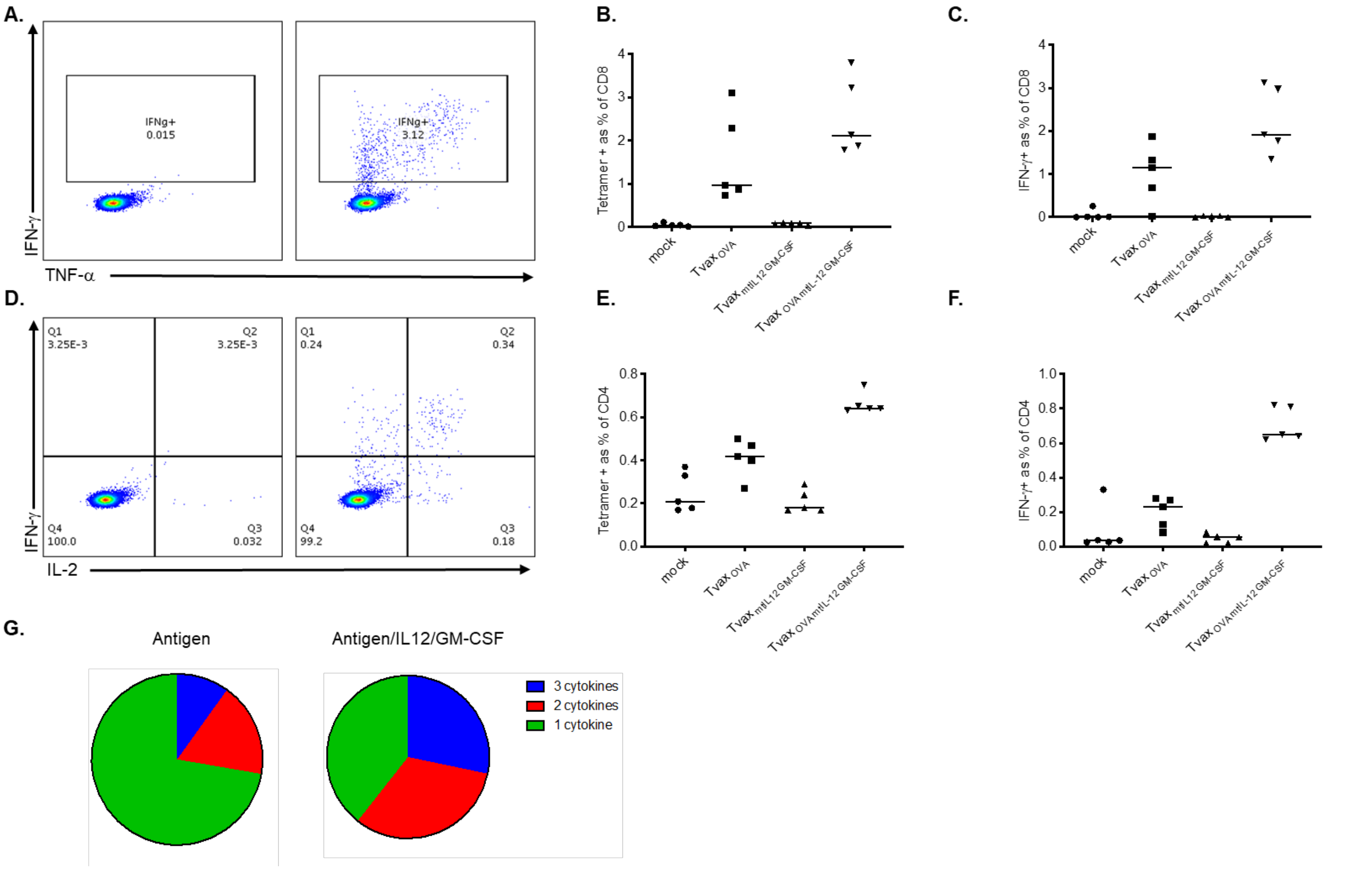

Fig. S6. Tvax induces polyfunctional T cell responses.

Splenocytes from mice vaccinated with antigen only (Tvax$_{Ova\ LLO}$) or mtIL-12 and GMCSF without antigen (Tvax$_{mtIL\text{-}12/GM\text{-}CSF}$) or with antigen (Tvax$_{Ova\ LLO/mtIL\text{-}12/GM\text{-}CSF}$) were harvested at day 13, stimulated with Ova and LLO190 peptides and analyzed for cytokine production by intracellular cytokine staining.

A. Representative intracellular staining of mock vaccinated mice (left panel) and Ova peptide (right panel) stimulated CD8 T cells for IFN-γ and TNF-α. The percentage of Ova specific CD8+ T cells in unstimulated splenocytes was determined by tetramer staining (B), and the percentage of IFN-γ producing cells following Ova peptide restimulation was measured in the same animals (C). D. Representative intracellular staining of mock vaccinated mice (left panel) and LLO190 peptide stimulated CD4 T cells for IFN-γ and IL-2. The percentage of LLO190 specific CD4+ T cells in unstimulated splenocytes was determined by tetramer staining (E), and the percentage of IFN-γ producing CD4+ cells following LLO190 peptide restimulation was measured in the same animals (F). G. The fraction of CD4+ T cells producing multiple cytokines following LLO190 stimulation out of IL-2, IFN-γ and TNF-α as a fraction of CD4+ T cells making at least one cytokine.

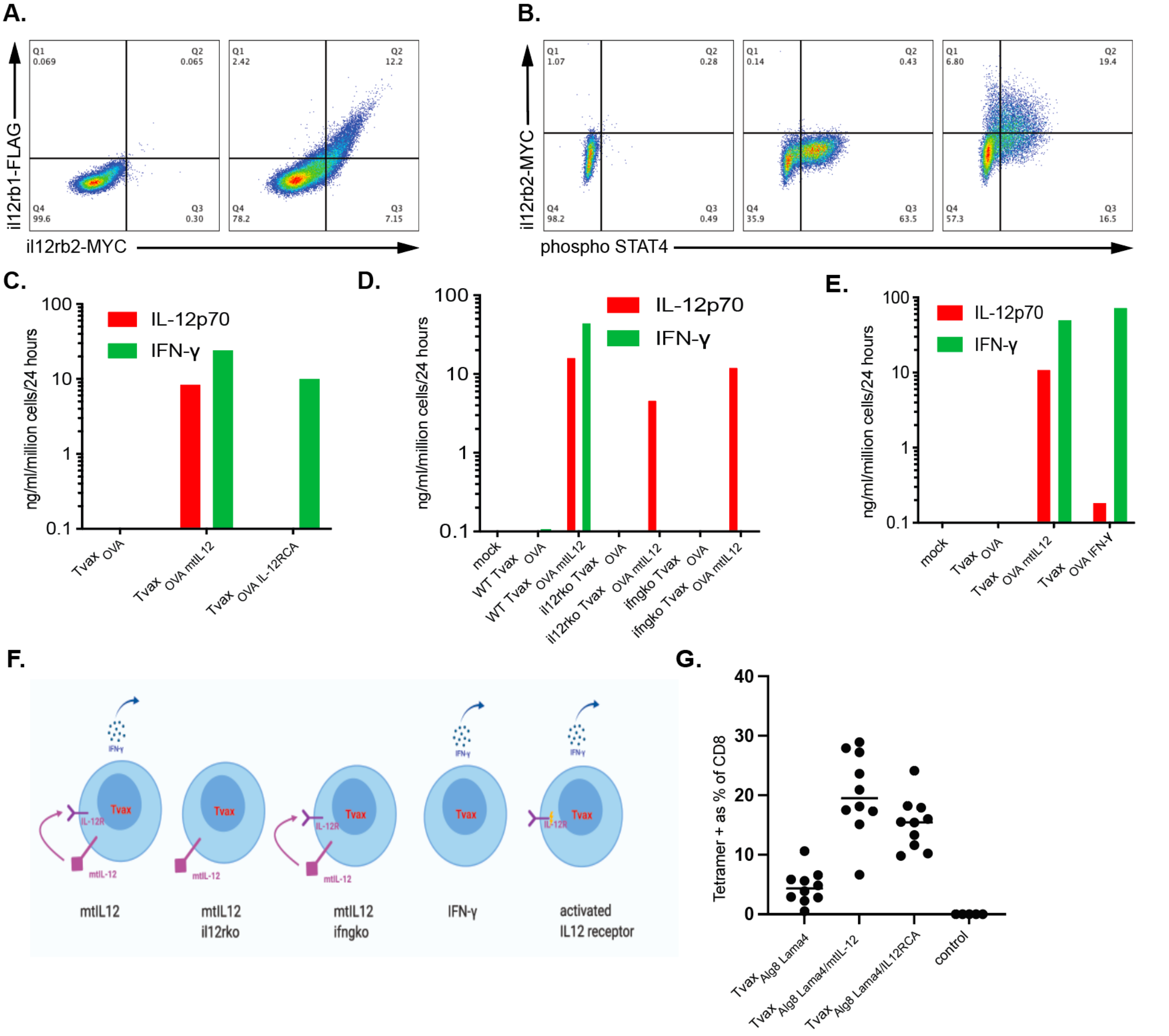

Fig. S7. **Expression of IL-12R$_{CA}$ or IFN-γ in Tvax leads to autocrine IFN-γ production.**
A. Tvax cells were transduced with tagged versions of IL-12R$_{CA}$ and expression of each subunit of the receptor was measured by flow cytometry using anti-Flag and anti-Myc antibodies. B. Staining for phosphorylated STAT4 in untransduced T cells and T cells transduced with mtIL-12 and IL-12R$_{CA}$. C-E. Secretion of IL-12p70 and IFN-γ by Tvax cells transduced with the indicated constructs measured over the 24 hours. F. Schematic of IFN-γ production by Tvax expressing each construct. G. Tvax expressing the Alg8 neoantigen alone or in combination with mtIL-12 or IL-12R$_{CA}$ were injected into recipient mice and the frequency of Alg8 specific CD8+ T cells were measured in the peripheral blood on day 8 by tetramer staining.

A.

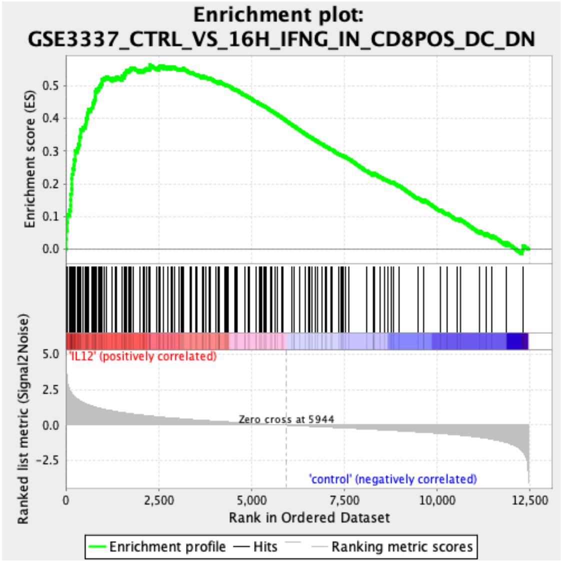

B.

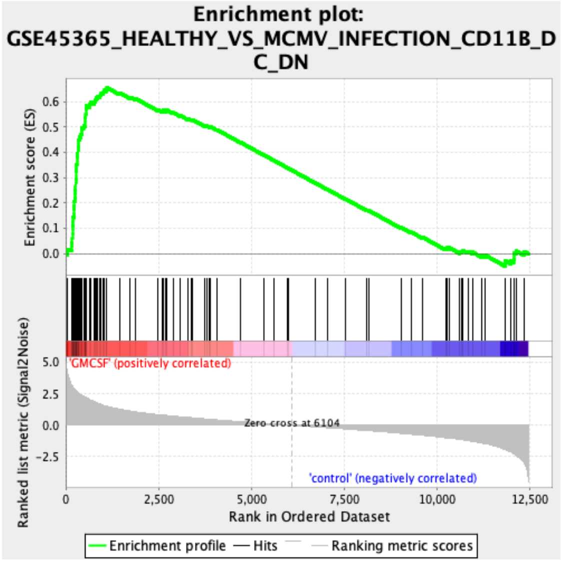

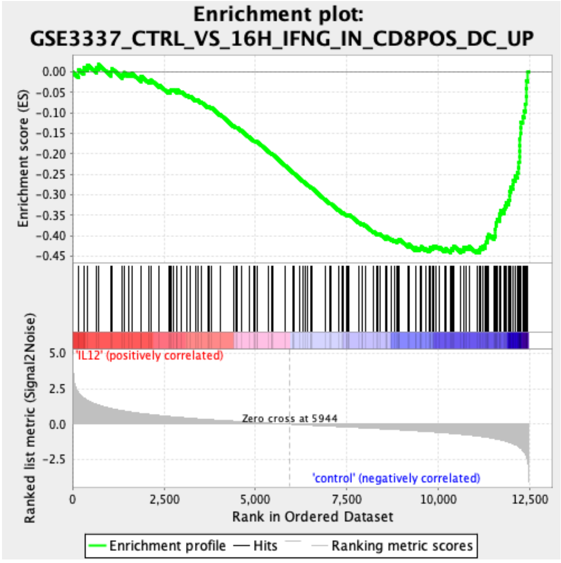

Fig. S8. Gene set enrichment analysis of exposure to Tvax with mtIL-12 and GM-CSF.
Tvax or Tvax$_{\text{mt-IL-12/GM-CSF}}$ without antigen were labelled with the lipophilic dye DiI, administered to mice and splenic DC were isolated 48 house after transfer for analysis (n=4 mice/group). Gene set enrichment analysis of genes upregulated and downregulated with mtIL-12 was compared to published data sets of IFN-γ treatment of DC (A) and GM-CSF were compared to MCMV infection (B).

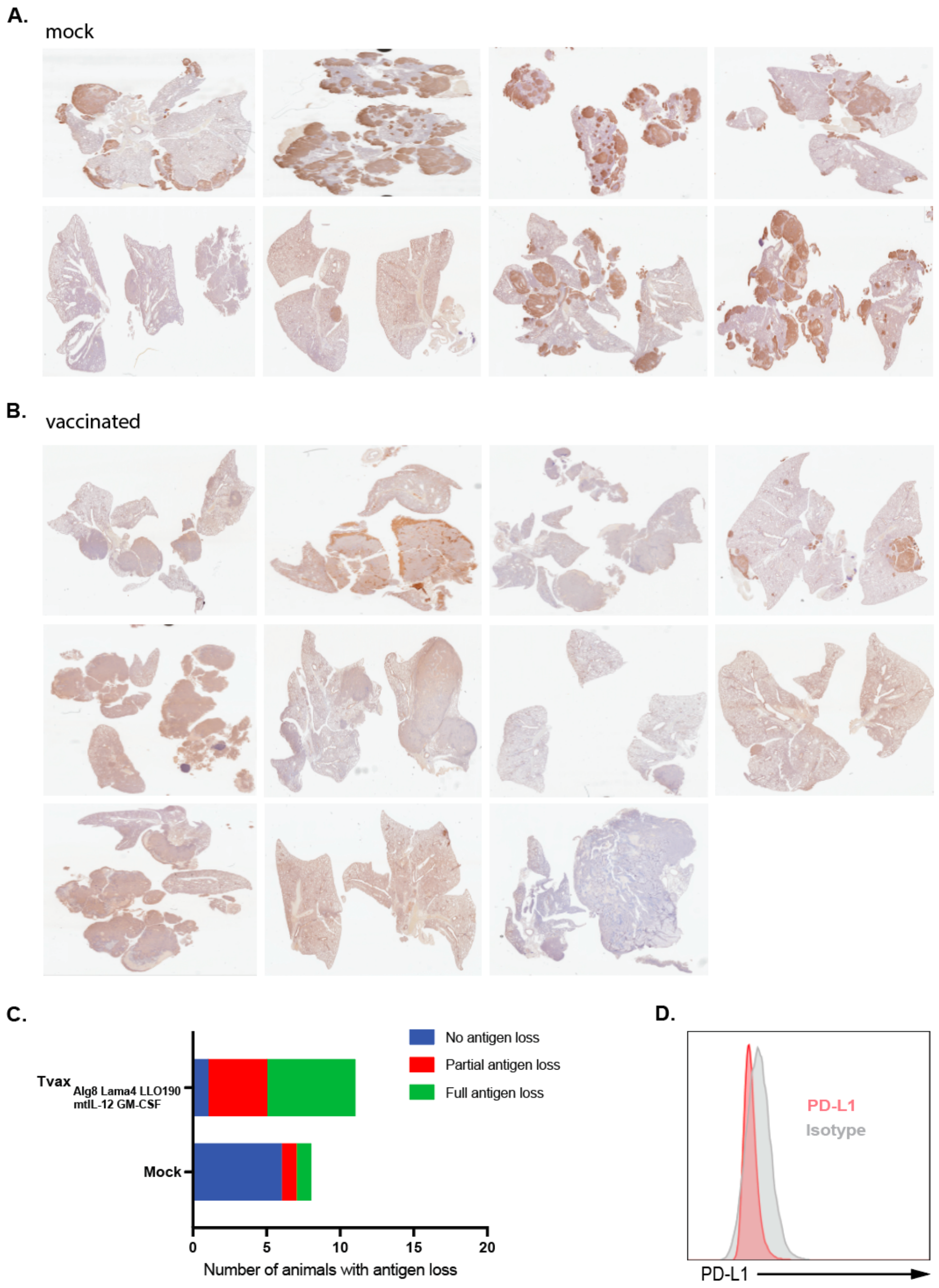

Fig. S9. Escape from Tvax treatment in lung metastasis model through antigen loss.
A-C. Mice were injected intravenously with B16-GFP-Alg8-Lama4-LLO190 and 4 days later vaccinated with a mock vaccine (A) or $\mathrm{Tvax}_{\mathrm{Alg8\ Lama4\ LLO/GM\text{-}CSF/mtIL\text{-}12}}$ (B). Lungs were harvested and stained for GFP when animals reached euthanasia criteria. C. summary of the number of animals with tumors identified on sections that partially or completely lost GFP expression. D. Human T cells modified by piggybac transposon, magnetically enriched and expanded were stained with an antibody to PD-L1 or an isotype control.
